# Impaired glucose tolerance and mild diabetes induce β-cell dysfunction in mice

**DOI:** 10.1038/s41467-026-71528-3

**Published:** 2026-04-30

**Authors:** Elizabeth Haythorne, Matthew Lloyd, Chris A. Smith, Martijn van de Bunt, Maria Rohm, Alice Elphick, Malgorzata Cyranka, Fiona M. Gribble, Frank Reimann, Frances M. Ashcroft

**Affiliations:** 1 Department of Physiology Anatomy and Genetics, Oxford, UK; 2 Institute for Neuroscience and Cardiovascular Research, University of Edinburgh, The Queen’s Medical Research Institute, Edinburgh BioQuarter, Edinburgh, UK; 3 Institute of Metabolic Science, University of Cambridge, Addenbrooke’s Hospital, Cambridge, UK; 4 Cytoki Pharma, Copenhagen, Denmark; 5 Institute for Diabetes and Cancer, Helmholtz Munich, Munich, Germany

**Keywords:** Type 2 diabetes, Energy metabolism

## Abstract

Severe chronic hyperglycaemia ( > 15 mM) causes impaired glycolytic and mitochondrial metabolism in pancreatic β-cells, leading to dramatically reduced insulin secretion and content. However, patients with type 2 diabetes often experience many years of reduced β-cell function and impaired glucose tolerance preceding diabetes diagnosis. It is postulated that β-cell function may be compromised by relatively small changes in glycaemia, initiating a gradual decline that underlies diabetes progression. We therefore investigated the extent to which impaired glucose tolerance and chronic mild hyperglycaemia are detrimental to β-cells. We show that chronic elevation of blood glucose of just 2-3 mM is sufficient to impair β-cell function, causing marked changes in metabolic gene expression and reducing insulin content, metabolic enzyme activity, mitochondrial oxidative phosphorylation and insulin secretion. Smaller but significant changes are produced by impaired glucose tolerance. These findings demonstrate that altered β-cell metabolism is an early event in type 2 diabetes development and highlight a need for therapeutic intervention during prediabetes.

## Abstract (web summary)

Haythorne et al. report that impaired glucose tolerance and mild hyperglycaemia cause dose-dependent changes in gene expression that further impair β-cell oxidative metabolism and insulin secretion in mice, and are likely to predispose to diabetes.

## Introduction

Diabetes is one of the leading causes of death and disability globally. In 2021, type 2 diabetes (T2D), the most common form of the disease, affected over 500 million people, a number that is projected to increase to over 1.3 billion by 2050^1^. The disease exacts a severe toll on the individual as the elevated blood glucose leads to secondary complications such as retinopathy, neuropathy, renal dysfunction and both micro- and macrovascular disease. The global economic costs are also substantial and the ability of governments to provide adequate healthcare facilities for affected individuals is challenging. A better understanding of the disease and its development is therefore essential.

T2D results from insufficient insulin secretion from pancreatic β-cells. As insulin is the only hormone capable of lowering blood glucose, a deficit leads to elevated fasting blood glucose concentrations and larger excursions following glucose intake. The disease has a strong genetic component but usually only develops in later life, the risk being enhanced by stressors such as age, obesity, and pregnancy. It is a progressive disorder that begins with impaired glucose tolerance (IGT, characterised by normal fasting glucose but an impaired response to a glucose challenge) and advances to diabetes as β-cell function gradually fails. By the time of diagnosis, it is estimated that as much as 50% of β-cell function has already been lost^2^. Understanding the molecular mechanisms that drive impaired β-cell function during IGT is therefore essential in order to develop preventative therapies.

There is accumulating evidence that impaired β-cell metabolism drives the progressive decline in insulin secretion in T2D. Marked changes in mitochondrial metabolism and in metabolic gene and protein expression have been identified in islets isolated from rodent models of diabetes^3–9^, in human islets cultured at high glucose^10–12^ or isolated from donors with type 2 diabetes^13–17^, and in β-cell lines exposed to chronic hyperglycaemia^7,18^. Multiple genes and proteins involved in glycolysis and gluconeogenesis are upregulated, whereas many of those involved in the tricarboxylic acid (TCA) cycle and the electron transport chain (ETC) are downregulated. This leads to a reduction in oxidative glucose metabolism, with glucose-induced increases in NAD(P)H, oxygen consumption and the ATP/ADP ratio all being impaired^5,7,9,15,19^.

In addition, bottlenecks in metabolism occur at both glyceraldehyde 3-phosphate dehydrogenase (GAPDH) and pyruvate dehydrogenase (PDH)^9,20^. The marked inhibition of GAPDH activity, despite a substantial increase in protein expression, likely results from a reduction in the essential cofactor NAD^+^, coupled with overproduction of NADH^7,21^. Reduced GAPDH activity will decrease downstream glycolysis, and substrate entry into the TCA cycle will be further reduced by elevation of pyruvate dehydrogenase kinase 1 (PDK1), which phosphorylates and inhibits PDH^9^. Reduced GAPDH activity also impairs glucose-stimulated insulin secretion in stem-cell derived β-cells^22,23^. Glycogen accumulation, a further indicator of impaired glucose metabolism, occurs in both human and rodent diabetic β-cells^5,24–26^. Glycogen is not normally found in β-cells^26^ as this would impede the ability of the cell to dynamically alter insulin release in response to changes in circulating blood glucose levels.

It has been argued that the response of the β-cell to chronic hyperglycaemia is a gradual process and that a relatively moderate increase in blood glucose reduces β-cell function, leading to less insulin secretion^9,27,28^. This elevates blood glucose further, thereby further decreasing insulin secretion. In this way, a vicious spiral develops in which the decline in β-cell function escalates exponentially. A key question that remains to be answered is whether there is a tipping point above which elevation of blood glucose precipitates this vicious spiral, and whether a relatively small increase in glycaemia is able to do so. The answer to this question has implications for the management of T2D.

Here, we investigated how high glucose must be elevated to initiate the changes in glucose metabolism and gene expression that drive β-cell decline. We show that changes in gene expression are precipitated by a small, chronic elevation of glucose and that the extent of these changes varies with the glucose concentration. We also provide evidence that impaired glucose tolerance or intermittent periods of hyperglycaemia are sufficient to impair β-cell function. Our results provide further support for the idea that progressive impairment of β-cell metabolism, induced by increasing hyperglycaemia, speeds T2D development.

## Results

To explore the effects of chronic hyperglycaemia on β-cell function, we used two models. First, the insulin-secreting cell line INS-1 832/13 (INS-1 cells), cultured at different glucose (G) concentrations for 48 h to simulate euglycaemia (5 mM glucose), mild diabetes (8 or 11 mM glucose) and severe diabetes (16 or 25 mM glucose). We term these cells 5G-, 8G-, 11G-, 16G- and 25G-cells. Secondly, we used islets isolated from hyperglycaemic βV59M mice^29^ with different free-fed blood glucose concentrations. The β-cell changes found in diabetic βV59M mice are prevented by restoration of euglycaemia with insulin, indicating they are due to hyperglycaemia/hypoinsulinaemia not K_ATP_ channel activation per se^29^.

### Effects of mild and severe chronic hyperglycaemia on INS-1 cells

**Fig. 1:**
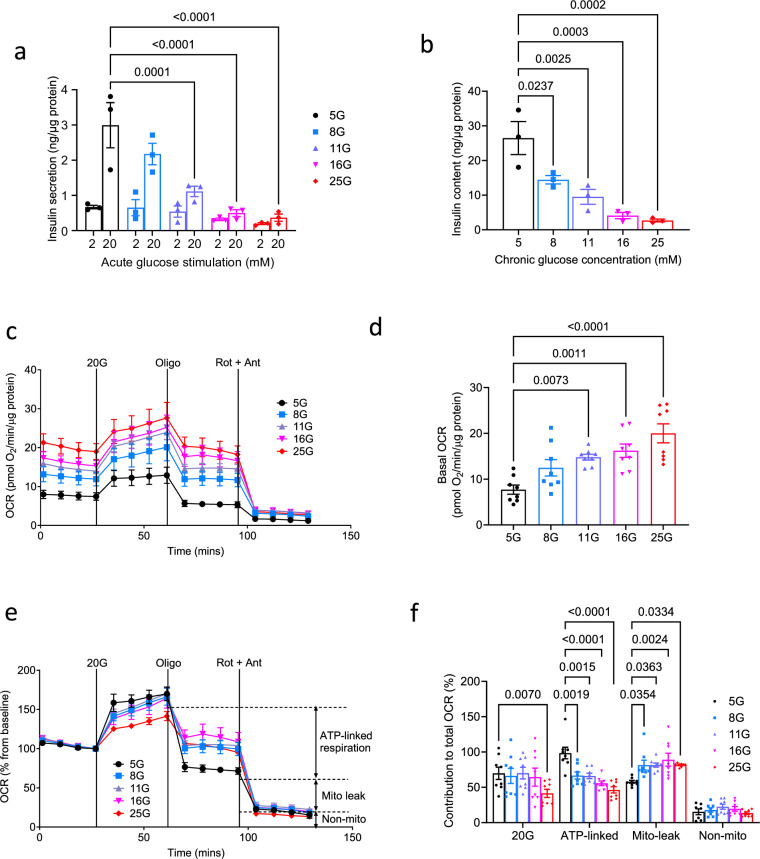
Effects of chronic glucose on insulin secretion and metabolism in INS-1 cells. **a**, **b** Insulin secretion at 2 and 20 mM glucose (**a**) and insulin content (**b**) in INS-1 cells cultured for 48 h at 5, 8, 11, 16 and 25 mM glucose (G) (*n* = 3). **c** Oxygen consumption rate (OCR) of INS-1 cells, cultured for 48 h at the indicated glucose concentrations, measured at 2 mM glucose and after sequential addition of 20 mM glucose (20 G), 1 μM oligomycin (Oligo) and 0.5 μM rotenone + 0.5 μM antimycin A (Rot+Ant) (*n* = 8). **d** Basal OCR at 2 mM glucose. Same data as in (**c**). **e** OCR expressed as the percentage change from baseline (at 2 mM glucose) and after sequential addition of 20 mM glucose (20 G), 1 μM oligomycin (Oligo) and 0.5 μM rotenone + 0.5 μM antimycin A (Rot+Ant) (*n* = 8). **f** Percentage change in OCR when glucose was raised from 2 mM to 20 mM (20 G), after oligomycin was applied (ATP-linked) after rotenone and antimycin A were applied (mito-leak) and the remaining non-mitochondrial OCR (non-mito). Same data as in (**e**). All panels show individual data points and mean ± s.e.m. Two-way ANOVA with Bonferroni post hoc test (**a**, **f**) and one-way ANOVA with Bonferroni post hoc test (**b**, **d**). *N* values indicate the number of biologically independent experiments. Source data are provided as a Source Data file.

To determine the extent to which ambient glucose must be elevated to impair β-cell function, we first examined the effects of chronic culture at different glucose concentrations on INS-1 cells. As Fig. 1a shows, glucose-stimulated insulin secretion decreased progressively as glucose was elevated and chronic exposure to glucose concentrations of 16 mM (16 G) and above almost abolished secretion. There was a parallel reduction in insulin content with increasing glucose concentrations (Fig. 1b).

Insulin gene expression (*Ins1, Ins2*) decreased with the ambient glucose concentration in a similar way, with both *Ins1* and *Ins*2 showing maximal expression in 5G-cells, being reduced by >50% in 8G-cells and declining further at higher glucose concentrations (Supplementary Fig. 1). Likewise, downregulation of expression at 8 mM glucose and above (compared to 5 mM glucose) was observed for expression of the insulin gene transcription factors (β-cell identity genes) *MafA, Neurod1, Nkx6-1*, *Pax6* and *Pdx1* (Supplementary Fig. 1). *Txnip* regulates insulin gene expression via induction of miR-204, which targets and downregulates *MafA* and thus insulin gene expression^30^. The marked increase ( ~ 10-fold) in *Txnip* expression in 8G-cells is consistent with the downregulation of *MafA*. These data demonstrate that chronic exposure of INS-1 cells to 8 mM glucose is sufficient to downregulate insulin gene expression and transcription factors controlling its expression.

We next examined the effects of chronic hyperglycaemia on both basal (at 2 mM) and glucose-stimulated (20 mM) oxygen consumption rate (OCR) (Fig. 1c–f). Basal OCR increased with increasing glucose concentration (Fig. 1c, d), as previously reported for human islets cultured for 4 days at 11 mM glucose^11^. However, glucose-stimulated OCR was unaffected until chronic glucose levels were elevated to 25 mM glucose, which reduced OCR by 50% (Fig. 1e, f). Glucose-stimulated OCR is composed of both ATP-linked (ATP-synthase activation) and non ATP-linked (mitochondrial leak) mitochondrial processes. ATP-linked respiration was significantly reduced and mitochondrial leak concurrently increased by as little as 8 mM glucose (Fig. 1f). The latter can explain both the lack of change observed in glucose-stimulated OCR and the increase in basal OCR. Thus, mitochondrial efficiency in β-cells is impaired by relatively small increases in chronic glucose exposure.

Glycolysis was also impaired by chronic hyperglycaemia, as indicated by attenuation of GAPDH activity, which began at 8 mM glucose and reached significance at 16 mM glucose (Supplementary Fig. 2a). GAPDH inhibition not only limits glycolytic flux but will also cause pooling of upstream glycolytic intermediates which, as shown previously in both 25G-cells and diabetic β-cells, is associated with constitutive inhibition of AMPK and hyperactivation of mTORC1^9,31–34^. Chronic culture for 48 h at 8 mM glucose was sufficient to markedly decrease AMPK activity when subsequently tested acutely at 2 mM glucose, and inhibition was complete in cells cultured at 16 mM glucose (Supplementary Fig. 2b,c). mTORC1 activity increased with increasing glucose concentration of the culture medium but did not become significant until glucose was elevated to 16 mM (Supplementary Fig. 2b,d). Thus, in INS-1 cells, AMPK appears more sensitive to chronic hyperglycaemia than mTORC1.

**Fig. 2:**
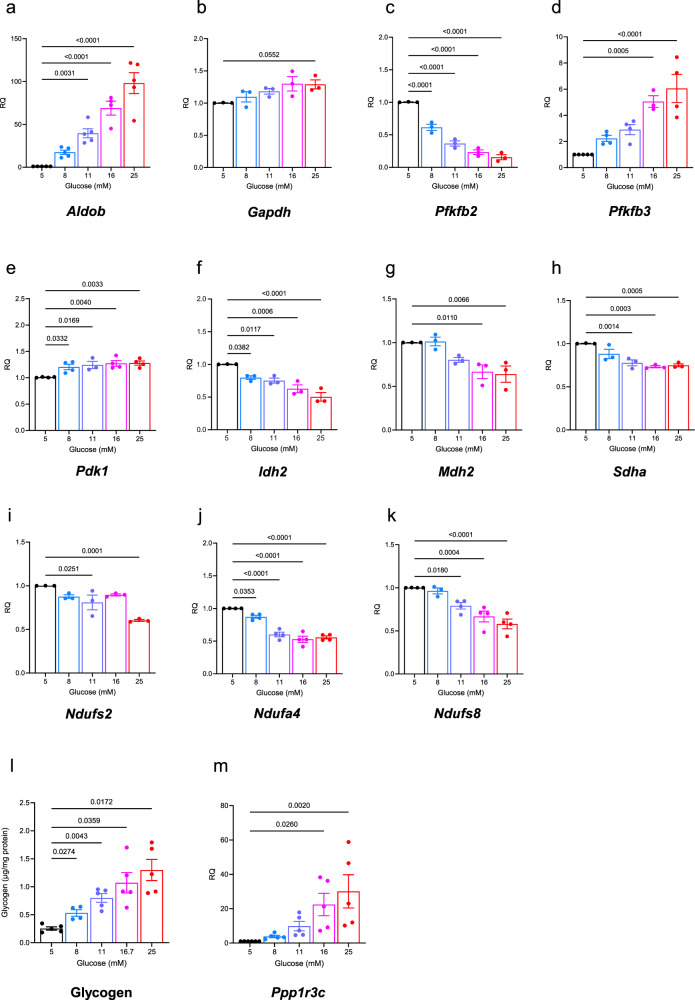
Effects of chronic glucose on glycogen and metabolic gene expression in INS-1 cells. **a**–**k** mRNA levels for the indicated genes in INS-1 cells cultured for 48 h at the indicated glucose concentrations. *n* = 3 for *Gapdh, Pfkfb2, Idh2, Mdh2, Sdha, Ndufs2*, *n* = 4 for *Pfkfb3, Pdk1, Ndufs8, Ndufa*4, and *n* = 5 for *Aldob*. **l**, **m** Glycogen content (**l**, *n* = 5, except for 8 mM glucose where *n* = 4) and mRNA levels of *Ppp1r3c* (**m**, *n* = 5) in INS-1 cells cultured for 48 h at the indicated glucose concentrations. Glycogen content is expressed as µg per mg protein. All panels show individual data points and mean ± s.e.m. One-way ANOVA with Bonferroni post hoc test. *N* values indicate the number of biologically independent experiments. Source data are provided as a Source Data file.

Activation of mTORC1 shifts glucose metabolism away from oxidative phosphorylation towards glycolysis, via transcriptional regulation of glycolytic genes^9,35^. Many glycolytic/gluconeogenic genes were very sensitive to chronic mild hyperglycaemia, including *Aldob, Gapdh, Pfkfb3* and *Pfkfb2*, with *Aldob* and *Pfkfb2* being markedly altered even in 8G-cells (Fig. 2a–d). We previously observed that culture at 25 mM glucose for 48 h downregulates most TCA cycle and many electron transport gene and proteins^7,9^. We now show (Fig. 2e–k) that for most genes tested changes in gene expression were also produced by chronic exposure to as little as 8 mM glucose (e.g. *Pdk1*, *Idh2, Ndufa4*) or 11 mM glucose (e.g. *Sdha, Ndufs2, Ndufs8*).

In addition to altered glycolytic and mitochondrial metabolism, a modest increase in chronic hyperglycaemia to 8 mM also caused significant elevation of β-cell glycogen content (Fig. 2l). Changes in glycogen content were paralleled by an increase in expression of *Ppp1r3c* (Fig. 2m), a glycogen-targeting subunit of protein phosphatase 1 that strongly promotes glycogen synthesis.

Taken together, these results demonstrate that chronic hyperglycaemia has deleterious effects on β-cell function, which are initiated by as little as 8 mM glucose and increase with increasing glucose concentrations.

### Effects of intermittent elevated glucose

**Fig. 3:**
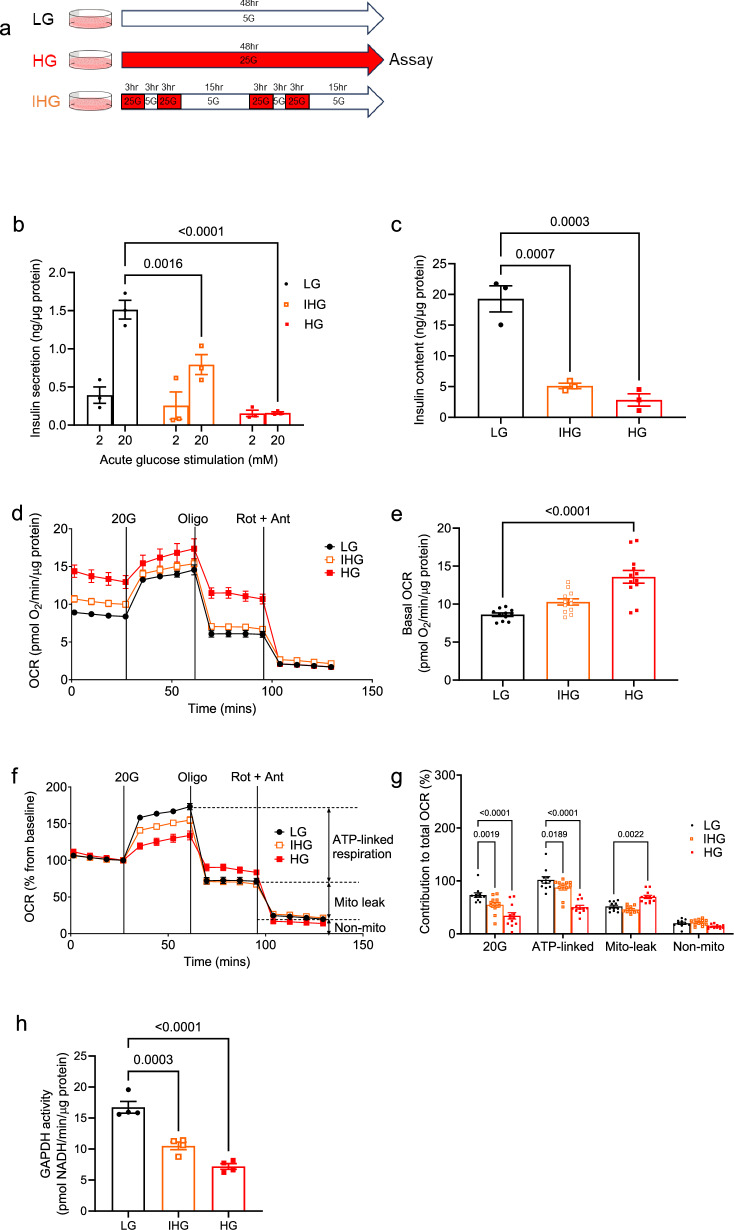
Effects of intermittent hyperglycaemia on INS-1 cells. **a** Experimental protocol for intermittent hyperglycaemia. INS-1 cells were cultured for 48 h either continuously at low glucose (LG: 5 mM) or high glucose (HG: 25 mM), or exposed intermittently to high glucose (IHG) for the times indicated. **b**, **c** Insulin secretion at 2 and 20 mM glucose and insulin content in INS-1 cells cultured for 48 h at LG (black), HG (red) or IHG (orange) (*n* = 3). **d** Oxygen consumption rate (OCR) of INS-1 cells cultured for 48 h either at LG (filled black circles), HG (filled red squares) or IHG (open orange squares), measured at 2 mM glucose and after sequential addition of 20 mM glucose (20 G), 1 μM oligomycin (Oligo) and 0.5 μM rotenone + 0.5 μM antimycin A (Rot+Ant) (LG cells, *n* = 11. HG and IHG cells, *n* = 12). **e** Basal OCR of INS-1 cells cultured for 48 h either continuously at LG (black), HG (red) or IHG (orange). Same data as in (**d**). **f** OCR expressed as the percentage change from baseline (at 2 mM glucose) and after sequential addition of 20 mM glucose (20 G), 1 μM oligomycin (Oligo) and 0.5 μM rotenone + 0.5 μM antimycin A (Rot+Ant) (LG cells, *n* = 11. HG and IHG cells, *n* = 12). **g** Percentage change in OCR when glucose was raised from 2 mM to 20 mM (20 G), after oligomycin application (ATP-linked), after rotenone and antimycin A application (mito-leak) and the remaining non-mitochondrial OCR (non-mito). Same data as in (**f**). **h** Glyceraldehyde 3-phosphate dehydrogenase (GAPDH) activity in INS-1 cells cultured for 48 h either continuously at LG (black), HG (red) or IHG (orange). (*n* = 4). All panels show individual data points and mean ± s.e.m. Two-way ANOVA with Bonferroni post hoc test (**b**, **g**) and one-way ANOVA with Bonferroni post hoc test (**c**, **e**, **h**). *N* values refer to biologically independent experiments. Source data are provided as a Source Data file.

In vivo, β-cells are exposed to frequent changes in circulating blood glucose concentrations, which rise after a meal and subsequently fall in response to insulin secretion. These excursions are greater and more prolonged in patients with impaired glucose tolerance^36–38^. To simulate this condition in vitro, we examined the effects of intermittent hyperglycaemia on insulin secretion. INS-1 cells were exposed to 25 mM glucose for 4 separate 3 h periods over the course of 48 h–a total of 12 h hyperglycaemia (Fig. 3a, IHG-cells). Despite a 15 h interval at 5 mM glucose before insulin secretion was examined, there was a marked reduction in both insulin secretion and insulin content of IHG-cells, albeit not as much as in HG-cells, which were continuously cultured for 48 h at 25 mM glucose (Fig. 3b, c). We also compared mitochondrial metabolism in IHG-cells and HG-cells (Fig. 3d–g). Intermittent hyperglycaemia had no significant effect on basal OCR. However, glucose-stimulated OCR and ATP-linked respiration were both significantly reduced, although not as much as in HG-cells. Mitochondrial leak and non-mitochondrial oxygen consumption were unaffected in IHG-cells, accounting for the unaltered OCR. Glycolytic metabolism was impaired in IHG-cells, as indicated by a significant decrease in GAPDH activity (Fig. 3h). These data indicate that impairments in β-cell function may accumulate during successive hyperglycaemic episodes and are not fully reversed by a 15 h recovery period at low glucose.

### Effects of varying plasma glucose concentrations in vivo

We next explored the effect of different levels of chronic hyperglycaemia using an in vivo model, the βV59M mouse. These mice selectively express an inducible activating K_ATP_ channel mutation (Kir6.2-V59M) in their β-cells that hyperpolarises the β-cell membrane potential and switches off insulin secretion following tamoxifen injection^29,39^. This leads to hypoinsulinaemia and hyperglycaemia. However, the mice are not obese, dyslipidaemic, or insulin resistant, enabling the effects of hyperglycaemia to be studied in isolation. The β-cell changes found in diabetic βV59M mice are prevented by restoration of euglycaemia with insulin, indicating they are due to hyperglycaemia/hypoinsulinaemia and not K_ATP_ channel activation per se^29^.

**Fig. 4:**
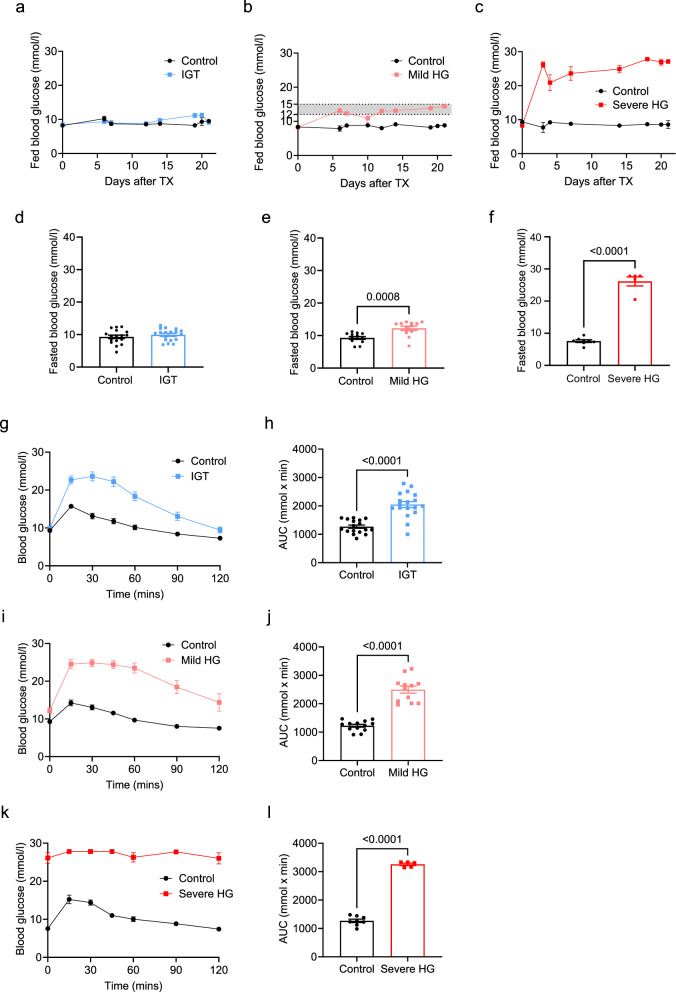
Regulation of glycaemia in mice with severe diabetes, mild diabetes and impaired glucose tolerance. Free-fed blood glucose levels in mice with **a** impaired glucose tolerance (IGT, *n* = 20) and corresponding controls (*n* = 21), **b** mild hyperglycaemia (mild-HG, *n* = 27) and corresponding controls (*n* = 31) or **c** severe hyperglycaemia (severe-HG, *n* = 15) and corresponding controls (*n* = 15), following tamoxifen injection at time zero. Control mice were injected with the same concentration of tamoxifen as their respective experimental cohorts. (d-f) Fasted blood glucose levels in (**d**) IGT mice (*n* = 18) and corresponding controls (*n* = 17), (**e**) mild-HG mice (*n* = 12) and corresponding controls (*n* = 13) and (**f**) severe-HG mice (*n* = 5) and corresponding controls (*n* = 8). **g**–**l** Glucose tolerance curves measured by IPGTT (1 g/kg body weight) and corresponding AUC measured in (**g**, **h**) IGT mice (*n* = 18) and controls (*n* = 17), **i**, **j** mild-HG mice (*n* = 12) and controls (*n* = 13) and (**k**, **l**) severe-HG mice (*n* = 5) and controls (*n* = 8). All panels show individual data points and mean ± s.e.m. Two-tailed unpaired Student’s *t* test. *N* values indicate the number of mice. Source data are provided as a Source Data file.

By injecting different amounts of tamoxifen, different blood glucose levels could be achieved in βV59M mice (Fig. 4a–c). Control animals (lacking the mutant gene) were injected with the same amount of tamoxifen. We grouped mice into 3 different categories of elevated plasma glucose: (i) those with free-fed blood glucose levels of >15 mM two weeks after tamoxifen injection (severe-HG); (ii) those with free-fed blood glucose levels between 12 and 15 mM glucose (mild-HG) and (iii) those with normal blood glucose levels ( ≤ 12 mM) but that showed impaired glucose tolerance (IGT) in a glucose tolerance test. We defined mild hyperglycaemia as a 2–3 mM rise above normal blood glucose.

Figure 4d–l shows glucose tolerance tests for IGT, mild-HG, and severe-HG mice and their respective controls. IGT mice had normal fasting and nearly normal 2 h blood glucose but showed substantially greater increases in blood glucose following a glucose challenge than wild-type mice (Fig. 4d, g). Mild-HG (Fig. 4e, i) and severe-HG (Fig. 4f, k) mice had elevated fasting and 2 hr blood glucose compared to their wild-type controls, with this difference being much greater for the severely diabetic mice. The area under the curve (AUC) was greater for all 3 experimental groups, increasing with disease severity (Fig. 4h, j, l).

### Gene expression analysis

It has been widely reported that chronic hyperglycaemia/diabetes alters the expression of multiple genes involved in glucose metabolism in both rodent and human islets^5–8,14,15,17^. Hence, we next examined changes in gene expression in islets isolated from control, IGT, mild-HG and severe-HG mice by RNAseq. There were substantially more significantly differentially expressed genes ( > 1.5 log_2_-fold change) in severe-HG islets (1862) and mild-HG (2029) islets compared with IGT islets (124) (Supplementary Data 1–3). PCA plots revealed that hyperglycaemic groups clearly pulled away from the control groups, whereas the IGT group was closer to the control mice (Supplementary Fig. 3).

KEGG analysis identified the TCA cycle as the most significant pathway affected in severe diabetes and multiple other metabolic pathways as being affected. Glycolysis, the pentose phosphate pathway, oxidative phosphorylation, pyruvate metabolism, and mTOR signalling were all within the top 20 (Supplementary Fig. 4). These pathways were also significantly affected by mild diabetes, consistent with the reduced oxidative metabolism observed in cell lines. Far fewer pathways were affected in IGT islets. However, pathways related to the lysosome, branched chain amino acid degradation, glycolysis, the pentose phosphate pathway and fructose and mannose metabolism were affected at all three stages of diabetes.

**Fig. 5:**
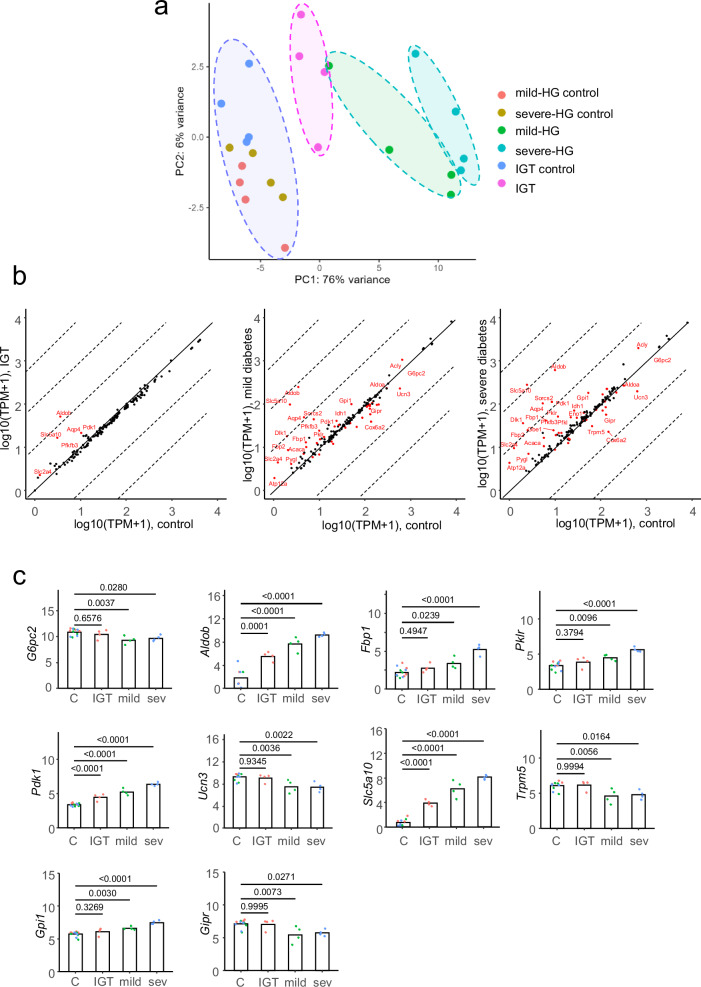
RNAseq in islets from in mice with severe diabetes, mild diabetes and impaired glucose tolerance. **a** PCA plot of RNAseq data from IGT, mild-HG and severe-HG mouse islets and their respective controls. The plot is based on metabolic genes only (see Supplementary Data 4). Control mice were injected with the same concentration of tamoxifen as their respective experimental cohorts. **b** log_10_-log_10_ plots of the average expression of metabolic genes in IGT, mild-HG and severe-HG mouse islets against their respective controls. The diagonal line indicates there is no difference in expression. Genes that are significantly differentially expressed are indicated in red, and those that are changed >1.5-fold are labelled. The dashed diagonal lines represent 10x, 100x, and 1000x enrichment in each direction. Genes located closer to the y-axis are enhanced in expression and those closer to the x axis show reduced expression. **c** Relative expression of selected genes (as indicated) in islets isolated from control (*n* = 12), IGT (red, *n* = 4), mild-HG (green, *n* = 4) and severe-HG (blue, *n* = 4) mice. Expression is normalised to that of the mean of all control samples and expressed on a log_2_ scale. Mean and individual data points are shown. Statistical tests were two-way ANOVA with diabetes severity a categorical variable, and Tukey’s HSD post-hoc test. N values in all panels refer to the number of mice. Source data are provided as a Source Data file.

As KEGG analysis revealed that metabolic pathways were those most affected by chronic hyperglycaemia, we focused on analysis of metabolic genes (see Methods), which resulted in clear separation of the transcriptome of islets from control, IGT and hyperglycaemic mice in the PCA plot generated using these metabolic genes (Fig. 5a, Supplementary Data 4). Figure 5b shows log_10_-log_10_ plots of the average expression of metabolic genes in IGT, mild-HG and severe-HG islets against their respective control islets. It is evident that there are large numbers of significantly differentially expressed genes (labelled red) in both severe-HG and mild-HG islets and many fewer in IGT-islets. In general, the magnitude and number of changes in gene expression were similar in mice with mild and severe diabetes (Supplementary Data 1,2), despite the very large difference in their random fed blood glucose (14.4 ± 0.3 mM v. 27.5 ± 0.3 mM, mean ± s.e.m.). Gene expression changes in IGT mice were relatively smaller and often did not reach significance. Among the most upregulated genes in all three experimental groups were *Aldob, Slc5a10, Aqp4, Pfkfb3, Slc2a4* and *Pdk1*: the latter is of particular significance as it will negatively regulate substrate entry into the TCA cycle (Fig. 5b, c and Supplementary Data 1-3). Mild diabetes also caused significant increases in expression of glycolytic genes (e.g. *Aldob, Pfkl, Fbp1, Fbp2, Pfkfb3, Gpi1, Eno1, Pdk1* and genes involved in fatty acid metabolism (*Acaca, Aacs, Acly*), as well as reduced expression of mitochondrial genes in both the TCA cycle and electron transport chain (e.g. *Idh2, Sdha, Mdh2, Cox6a2*) (Fig. 5b, c and Supplementary Data 1–3). Glucose-6-phosphatase (*G6pc2*) was reduced and *Ppp1r3c* increased in both mild-HG and severe-HG islets (Fig. 5b, c and Supplementary Data 1-3), which is predicted to increase glycogen levels (as it does in INS-1 cells and islets)^5,40^.

Numerous other genes that play important roles in insulin secretion were also altered (Supplementary Data 1,2). For example, expression of the GIP receptor gene (*Gipr*) was downregulated in mild-HG and severe-HG islets, whereas expression of the glucagon receptor (*Gcgr*) was upregulated (Fig. 5c, Supplementary Data 1,2). We also observed reduced expression of the non-selective cation channel *Trpm5* (Fig. 5 b,c and Supplementary Data 1,2). Polymorphisms in *TRPM5* enhance T2D risk in humans^41^. Likewise, *Pparg*, which was downregulated in severe-HG islets (Supplementary Data 1-3), is associated with T2D^42^. PPARG regulates transcription of genes involved in glucose sensing, insulin secretion and insulin gene transcription^43^. Variants in *CDKAL1*, which was upregulated in severe-HG islets (Supplementary Data 1–3), are also linked to an increased risk of T2D^44^. *Ucn3*, which is co-secreted with insulin, was markedly downregulated (Fig. 5c). Finally, we observed that certain genes implicated in β-cell ‘de-differentiation’ (e.g., *Foxa2, Aldh1a3*^45^,) were affected in both mild-HG and severe-HG islets (Supplementary Data 1,2).

**Fig. 6:**
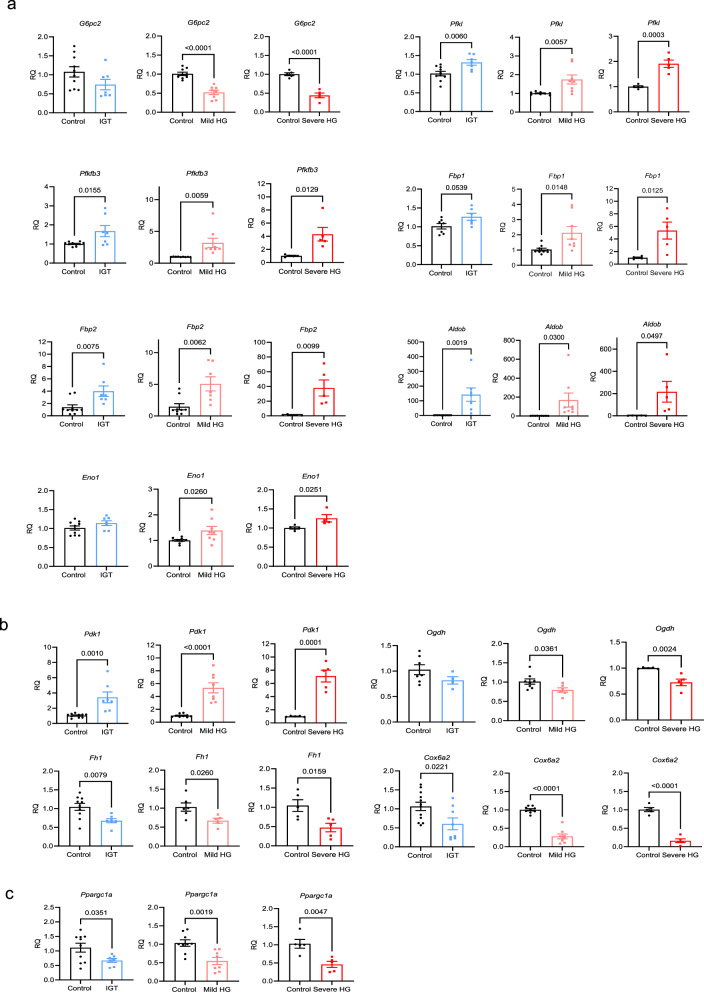
Metabolic gene expression in mice with severe diabetes, mild diabetes and impaired glucose tolerance. mRNA levels, measured by qPCR, for the indicated genes in islets isolated from mice with impaired glucose tolerance (IGT, blue), mild hyperglycaemia (mild-HG, pink) and severe hyperglycaemia (severe-HG, red) and their respective controls (black). Control mice were injected with the same concentration of tamoxifen as their respective experimental cohorts. **a**–**c** mRNA levels of the indicated genes involved in **a** glycolytic/gluconeogenic, **b** mitochondrial and **c** glycogen metabolism. *G6pc2* (*n* = 7 IGT, *n* = 10 controls; *n* = 8 mild HG, *n* = 9 controls; *n* = 5 severe HG, *n* = 5 controls), *Pfkl* (*n* = 7 IGT, *n* = 10 controls; *n* = 8 mild HG, *n* = 9 controls; *n* = 5 severe HG, *n* = 5 controls), *Pfkfb3* (*n* = 7 IGT, *n* = 10 controls; *n* = 8 mild HG, *n* = 9 controls; *n* = 5 severe HG, *n* = 5 controls), *Fbp1* (*n* = 6 IGT, *n* = 7 controls; *n* = 8 mild HG, *n* = 9 controls; *n* = 5 severe HG, *n* = 5 controls), *Fbp2* (*n* = 7 IGT, *n* = 10 controls; *n* = 7 mild HG, *n* = 9 controls; *n* = 5 severe HG, *n* = 5 controls), *Aldob* (n = 7 IGT, *n* = 10 controls; *n* = 8 mild HG, *n* = 9 controls; *n* = 5 severe HG, *n* = 5 controls), *Eno1* (n = 7 IGT, *n* = 10 controls; *n* = 8 mild HG, *n* = 9 controls; *n* = 4 severe HG, *n* = 5 controls) *Pdk1* (n = 7 IGT, *n* = 10 controls; *n* = 8 mild HG, *n* = 9 controls; *n* = 5 severe HG, *n* = 5 controls), *Ogdh* (n = 4 IGT, *n* = 7 controls; *n* = 6 mild HG, *n* = 8 controls; *n* = 5 severe HG, *n* = 5 controls), *Fh1* (n = 7 IGT, *n* = 10 controls; *n* = 5 mild HG, *n* = 6 controls; *n* = 5 severe HG, *n* = 5 controls), *Cox6a2* (*n* = 8 IGT, *n* = 12 controls; *n* = 8 mild HG, 9 controls; *n* = 5 severe HG, *n* = 5 controls), *Ppargc1a* (*n* = 7 IGT, *n* = 10 controls; *n* = 8 mild HG, *n* = 9 controls; *n* = 5 severe HG, *n* = 5 controls). All panels show individual data points and mean ± s.e.m. Two-tailed unpaired Student’s *t* test. *N* values refer to the number of mice. Source data are provided as a Source Data file.

We next examined expression of selected genes by qPCR (Fig. 6a, b). This largely recapitulated what was found by RNAseq, with glycolytic and gluconeogenic genes being upregulated and TCA and ETC genes being downregulated. In most cases, the more severe the diabetes the greater the change. *Pfkl, Pfkfb3, Fbp2, Aldob, Pdk1, Fh1* and *Cox6a2* were especially sensitive to changes in blood glucose, expression being markedly affected even in IGT-islets (Fig. 6a, b). *Ppargc1a*, a transcriptional coactivator involved in mitochondrial biogenesis and expression of nuclear-encoded mitochondrial proteins, was downregulated in IGT, mild-HG and severe-HG mouse islets in qPCR studies but was not significantly changed in the RNAseq data (Fig. 6c and Supplementary Data 1–3). *Ppargc1a* was also downregulated in severe-HG islets^7^ and in islets from donors with T2D^46^. *G6pc2* and *Eno1* were altered in mild-HG and severe-HG, but not IGT, indicating they are less sensitive to transient increases in blood glucose.

### Effects of different glucose levels on β-cell metabolic enzymes

**Fig. 7:**
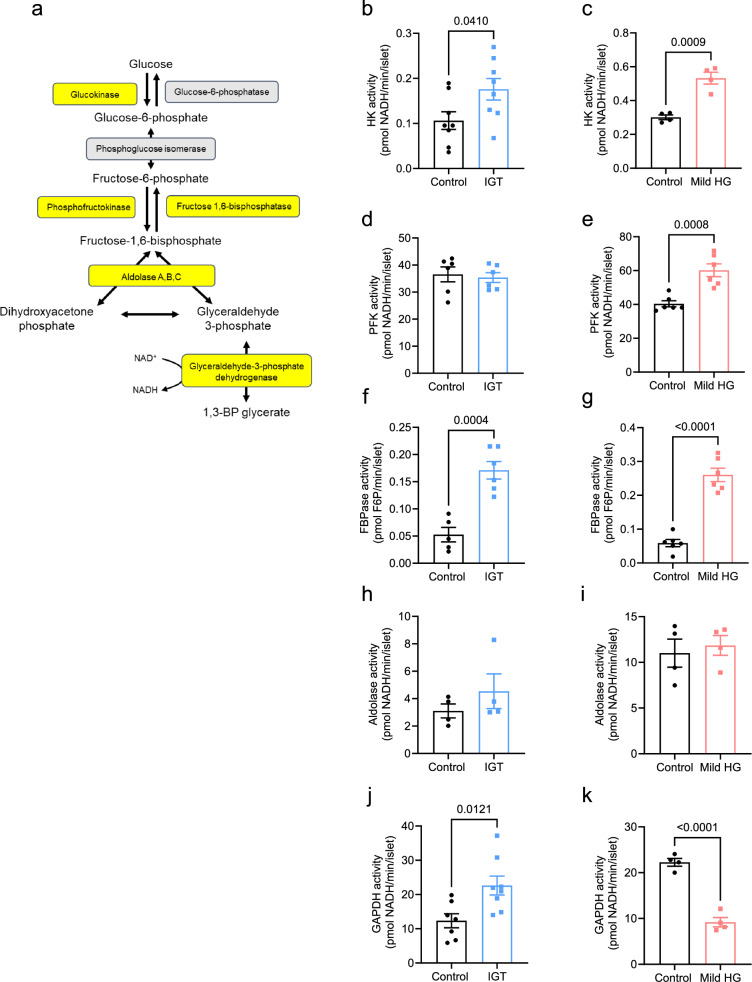
Changes in metabolic enzyme activity induced by chronic hyperglycaemia. **a** Schematic of glycolysis. Enzymes whose activity was measured are indicated in yellow. **b**–**k** Activity of the indicated glycolytic/gluconeogenic enzymes in islets from mice with impaired glucose tolerance (IGT, blue) or mild hyperglycaemia (mild-HG, pink) and their corresponding controls (black). Control mice were injected with the same concentration of tamoxifen as their respective experimental cohorts. **b**, **c** Hexokinase (HK) activity in: **b** IGT mice (*n* = 8) and controls (*n* = 8); **c** mild-HG mice (*n* = 4) and controls (*n* = 4). **d**, **e** Phosphofructokinase (PFK) activity in **d** IGT mice (*n* = 6) and controls (*n* = 6); **e** mild-HG mice (*n* = 6) and controls (*n* = 6). **f**, **g** Fructose bisphosphatase (FBPase) activity in: (f) IGT mice (*n* = 6) and controls (*n* = 5); **g** mild-HG mice (*n* = 6) and controls(*n* = 6). Aldolase activity in: (**h**) IGT (*n* = 4) and control (*n* = 4) mice; **i** mild-HG (*n* = 4) and control (*n* = 4) mice. Glyceraldehydye 3-phosphate dehydrogenase (GAPDH) activity in: **j** IGT (*n* = 8) and control (*n* = 7) mice; **k** mild-HG (*n* = 4) and control (*n* = 4) mice. All experimental panels show individual data points and mean ± s.e.m. Two-tailed unpaired Student’s *t* test. *N* values refer to the number of mice. Source data are provided as a Source Data file.

The changes in metabolic gene expression we observed suggest that IGT and mild diabetes lead to changes in β-cell metabolism, as is the case for severe diabetes^7,9^. We therefore measured the activity of key glycolytic and gluconeogenic enzymes (Fig. 7a). Hexokinase activity (which in islets predominantly represents glucokinase activity) was increased in both IGT and mild-HG islets (Fig. 7b, c). Phosphofructokinase activity was unaltered in IGT-islets but increased in mild-HG islets (Fig. 7d, e). Activity of the gluconeogenic enzyme, fructose-1,6-bisphosphatase, was substantially increased in both IGT and mild-HG islets (Fig. 7f, g). However, the activity of aldolase was unaffected in both sets of mice (Fig. 7h, i). There was an increase in GAPDH activity in IGT islets (Fig. 7j), whereas mild-HG islets showed a substantial reduction in GAPDH activity (Fig. 7k), albeit less than that seen in severe-HG islets ( > 90%^9^).

### Effects of different glucose levels on β-cell oxidative metabolism

**Fig. 8:**
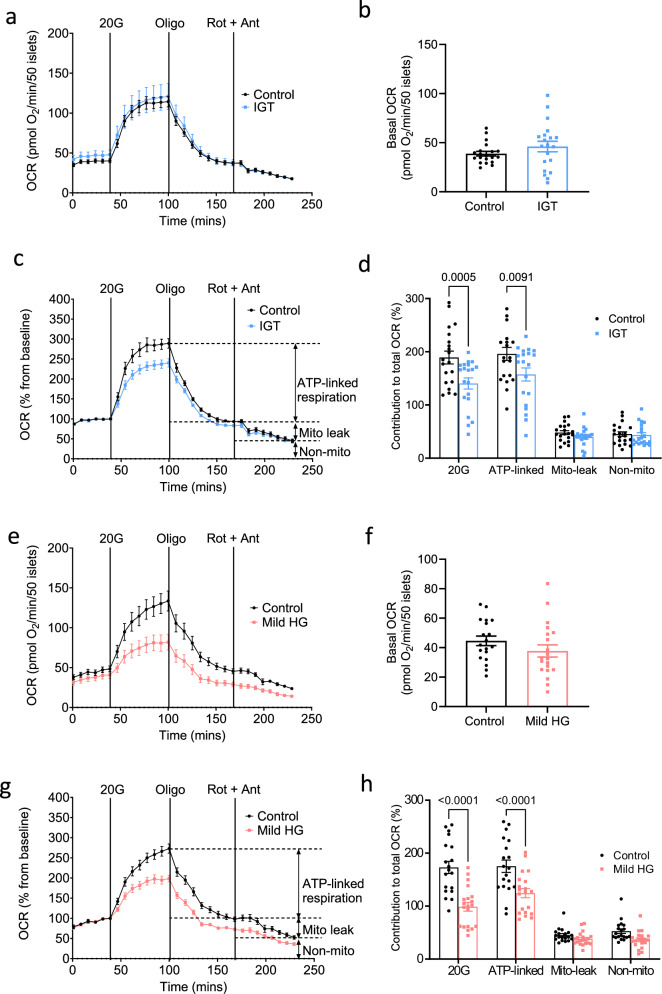
Mild hyperglycaemia impairs oxidative metabolism. Oxygen consumption rate (OCR) of islets from mice with **a** impaired glucose tolerance (IGT, blue) or **e** mild hyperglycaemia (mild-HG, pink) and their corresponding controls (black). OCR is shown at baseline (2 mM glucose) and after sequential addition of 20 mM glucose (20 G), 5 μM oligomycin (Oligo) and 5 μM rotenone + 5 μM antimycin A (Rot+Ant). **a** IGT islets (blue, *n* = 8 mice examined over 20 independent experiments) and controls (black, *n* = 8 mice examined over 20 independent experiments). **e** mild-HG islets (pink, *n* = 8 animals examined over 20 independent experiments) and control islets (black, *n* = 8 mice examined over 19 independent experiments). **b**, **f** Basal OCR at 2 mM glucose. **b** for IGT (blue) and control (black) islets. Same experiment as in (**a**). **f** for mild-HG (pink) and control (black) islets. Same experiment as in (**e**). **c**, **g** OCR of islets from mice with IGT (**c**) or mild-HG (**g**) and corresponding control mice expressed as the percentage change from the OCR baseline (at 2 mM glucose) and after sequential addition of 20 mM glucose (20 G), 5 μM oligomycin (Oligo) and 5 μM rotenone + 5 μM antimycin A (Rot+Ant). **c** IGT islets (blue, n = 8 mice examined over 20 independent experiments) and control islets (black, *n* = 8 mice examined over 20 independent experiments). **g** mild-HG islets (pink, *n* = 8 animals examined over 20 independent experiments). Control islets (black, *n* = 8 mice examined over 19 independent experiments). **d**, **h** Percentage change in OCR when glucose was raised from 2 mM to 20 mM (20 G), after oligomycin was applied (ATP-linked) after rotenone and antimycin A were applied (mito-leak) and the remaining non-mitochondrial OCR (non-mito). **d** Same data as in (**c**). **h** same data as in (**g**). All panels show individual data points and mean ± s.e.m. Two-way ANOVA with Bonferroni post hoc test. Source data are provided as a Source Data file.

Severe diabetes dramatically reduces the increase in both islet OCR and ATP-linked (oligomycin-sensitive) respiration produced by 20 mM glucose^9^. This was also the case for both IGT (Fig. 8a, c, d) and mild-HG islets (Fig. 8e, g, h), albeit less severely than seen previously in severe-HG islets. Mitochondrial leak and non-mitochondrial oxygen consumption were unaffected in all 3 experimental groups. However, whereas basal OCR (at 2 mM glucose) was strongly reduced in severe-HG islets^9^ it was unaffected in both IGT (Fig. 8b) and mild-HG (Fig. 8f) islets.

### Effects of NAD^+^ supplementation

**Fig. 9:**
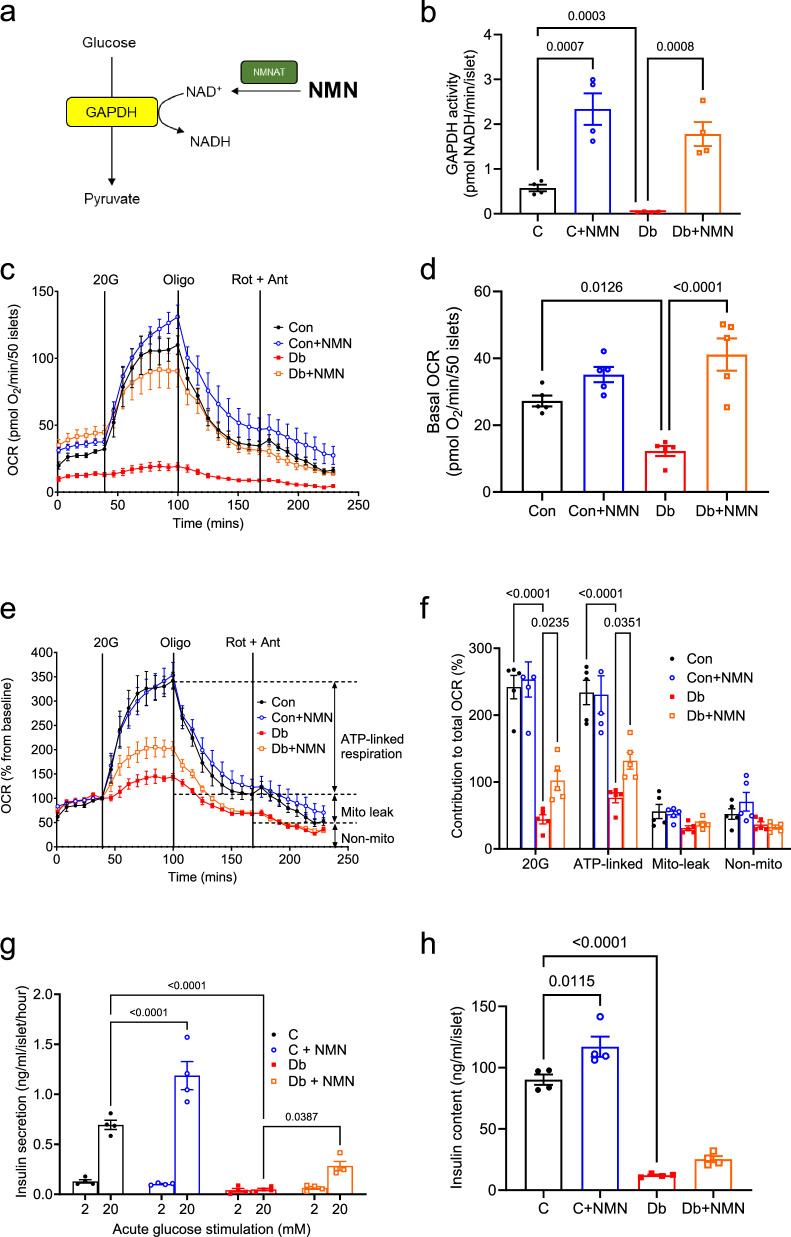
Augmentation of NAD^+^-biogenesis improves glucose metabolism and insulin secretion in diabetic islets. **a** Schematic of nicotinamide mononucleotide (NMN) conversion to NAD^+^, by nicotinamide mononucleotide adenylyltransferase (NMNAT). **b** GAPDH activity in islets from diabetic mice (Db, *n* = 4) and controls (C, *n* = 4) cultured for 48 h ± 100 µM NMN. **c** OCR in control (Con) and diabetic (Db) islets cultured for 48 h  ±  100 µM NMN. OCR is shown at baseline (2 mM glucose) and after sequential addition of 20 mM glucose (20 G), 5 μM oligomycin (Oligo) and 5 μM rotenone + 5 μM antimycin A (Rot+Ant). Control islets (black, *n* = 3 mice examined over 5 independent experiments), Control islets + 100 µM NMN (blue, *n* = 3 mice examined over 5 independent experiments), Diabetic islets (red, *n* = 3 mice examined over 5 independent experiments), Diabetic islets + 100 µM NMN (orange, *n* = 3 mice examined over 5 independent experiments). **d** Basal OCR at 2 mM glucose. Same experiment as in (**c**). **e** OCR in diabetic and control islets expressed as the percentage change from the OCR baseline (at 2 mM glucose) and after sequential addition of 20 mM glucose (20 G), 5 μM oligomycin (Oligo) and 5 μM rotenone + 5 μM antimycin A (Rot+Ant). Control islets (black, *n* = 3 mice examined over 5 independent experiments), Control islets + 100 µM NMN (blue, *n* = 3 mice examined over 5 independent experiments), Diabetic islets (red, *n* = 3 mice examined over 5 independent experiments), Diabetic islets + 100 µM NMN (orange, *n* = 3 mice examined over 5 independent experiments). **f** Percentage change in OCR when glucose was raised from 2 mM to 20 mM (20 G), after oligomycin was applied (ATP-linked) after rotenone and antimycin A were applied (mito-leak) and the remaining non-mitochondrial OCR (non-mito). Same data as in (**e**). **g** Insulin secretion and (**h**) insulin content from control (C) and diabetic (Db) islets incubated for 48 h  ±  100 µM NMN and then stimulated with 2 mM or 20 mM glucose for 1 h (*n*  =  5 animals/genotype). **b**–**h** Mean ± s.e.m. and individual data points are shown. One-way ANOVA with Bonferroni post hoc test (**b**, **d**, **h**) and two-way ANOVA with Bonferroni post hoc test (**f**, **g**). Significance against control data is indicated. Source data are provided as a Source Data file.

We hypothesised that the reduction in GAPDH activity in HG-cells and diabetic islets is due to insufficient NAD^+^, and that this underlies impaired glucose-stimulated mitochondrial respiration and insulin secretion. To test this idea, we cultured islets from control and severe-HG mice for 48 hrs ± 100 µM β-nicotinamide mononucleotide (NMN), which is membrane permeant and converted to NAD^+^ by the enzyme nicotinamide mononucleotide adenylyltransferase (NMNAT) (Fig. 9a). This markedly enhanced GAPDH activity in both groups (Fig. 9b). It also restored basal OCR and partially restored both glucose-stimulated and ATP-stimulated OCR (Fig. 9c-f). NMN supplementation potentiated glucose-stimulated insulin secretion and insulin content in both groups (Fig. 9g, h), similar to previous studies in human islets^47^.

### Effects of diabetes reversal

Finally, we examined if reversal of glycaemia could restore gene expression in diabetic mouse islets. Following 2 weeks of severe-HG we treated mice with glibenclamide, which closes the open K_ATP_ channels, thereby stimulating insulin secretion and normalising blood glucose^29^.

**Fig. 10:**
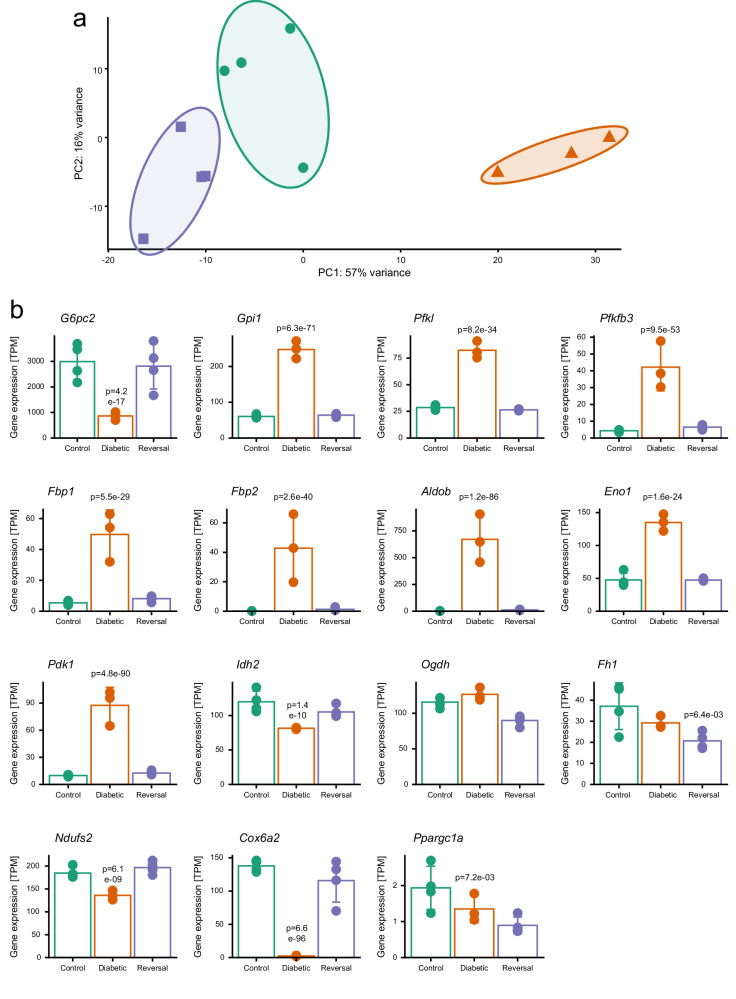
Restoration of euglycaemia reverses glucose-induced changes in gene expression. **a** PCA plot of RNAseq data from islets isolated from control (green, *n* = 4), severe-HG (red, *n* = 4) and glibenclamide-treated severe-HG (blue, *n* = 4) mice. The plot is based on the 500 most variable genes across all samples. **b** Expression of selected genes (as indicated) in islets isolated from control (green, *n* = 4), severe-HG (red, *n* = 3) and glibenclamide-treated (blue, *n* = 4) mice. Expression is shown as transcripts per million (TPM). Mean ( ± SD) and individual data points are shown. Significance against control data is indicated. Exact test for a difference in mean between two groups of negative binomial random variables as implemented in the R package *edgeR* with multiple testing correction using the Benjamini–Hochberg procedure. Source data are provided as a Source Data file.

Unbiased gene expression analysis of isolated islets using RNA sequencing shows that expression of many key metabolic genes was almost fully restored (Fig. 10)–only 342 genes remained significantly (FDR < 1%) different between islets isolated from control and reversal mice (Supplementary Data 5), and many of the most glucose-sensitive genes, including those that are altered in IGT mouse islets, were either fully or partially restored (Supplementary Data 5). We have also previously shown^5^ that culture of 4-week diabetic islets at 5 mM glucose (or 5 mM glucose plus 2 µM of the sulphonylurea gliclazide) for 72 h also restores glucose-stimulated NADH and ATP responses, indicative of normalised mitochondrial metabolism.

## Discussion

Our results provide insights into the molecular mechanisms underlying β-cell dysregulation and failure during the early stages of T2D. We show that detrimental changes in gene expression and glucose metabolism occur prior to the onset of sustained hyperglycaemia, during impaired glucose tolerance, and that almost all of the changes that occur in response to severe hyperglycaemia are recapitulated following only modest elevation of ambient glucose.

By injecting βV59M mice with different amounts of tamoxifen, we induced mild (12–15 mM glucose) and severe hyperglycaemia ( > 15 mM glucose) in adult mice. Additionally, we generated mice with normal free-fed and fasted blood glucose, but that exhibited impaired glucose tolerance, reminiscent of prediabetes. This approach enabled us to determine the effect of different blood glucose levels on β-cell function in the absence of obesity or insulin resistance and did not require invasive surgery or use of toxins. Injection of a lesser tamoxifen concentration is likely to result in activation of the K_ATP_ channel in fewer β-cells rather than a reduced activation in all β-cells. However, activation in fewer β-cells will result in an overall lesser reduction of insulin release and, as observed, a lesser elevation of blood glucose. Importantly, all β-cells will be exposed to the same circulating glucose concentration.

Most of the data on INS-1 cells and islets are in good agreement, despite the difference in species (rat versus mouse), duration of hyperglycaemia (48 h versus ~2 weeks), and the fact that β-cells in our diabetic islets will be hyperpolarised (due to the activating K_ATP_ channel mutation) whereas glucose will depolarise INS-1 cells. This argues that most of the changes we observe are due to the elevated extracellular glucose concentration. Any differences may, in part, be a consequence of an increased cytosolic calcium in INS-1 cells provoked by glucose-stimulated depolarisation. The main difference between the INS-1 cells and islet data is that chronic hyperglycaemia increased basal OCR in INS-1 cells but reduced or tended to reduce it in islets, depending on severity. This may be attributed to the increase in mitochondrial leak seen in INS-1 cells (but not islets).

Severe chronic hyperglycaemia produced dramatic changes in metabolism, metabolic enzyme activity and expression, insulin secretion and insulin content, as previously described for βV59M mice, for other diabetic mouse models and for diabetic human islets^3–9,13–17,48^. A very small, sustained increase in blood glucose, just 2-3 mM above normal, recapitulated these effects, increasing upper glycolysis (measured as glucokinase and phosphofructokinase activity) and gluconeogenesis (fructose bisphosphatase activity), but downregulating GAPDH activity, and glucose-stimulated mitochondrial respiration and coupling efficiency. Similar changes in the expression of many genes were also observed, with many of the most differentially regulated genes and pathways being those involved in metabolism. Strikingly, our data also show that impaired glucose tolerance (in mice) or intermittent hyperglycaemia (in INS-1 cells) is sufficient to initiate these changes in metabolic gene expression and metabolism. This argues that the reversal of the gene changes induced by hyperglycaemia on restoration of euglycaemia is slow—which might cause accumulating changes in gene expression even in mild diabetes or glucose intolerance.

More than 80% of glucose that enters the β-cell is normally metabolised by the mitochondria to ATP^49^, which ensures that insulin secretion is tightly coupled to changes in extracellular glucose. This explains why even small metabolic perturbations are detrimental to insulin secretion.

Many of the changes in metabolic enzyme activity that we observed in mild and severe diabetes were also present, albeit at a lesser level, in IGT islets. In particular, hexokinase and FBPase activity were enhanced and both glucose-stimulated and ATP-linked respiration were reduced. These changes in enzyme activity may be a consequence of the reduced expression of *G6pc2* and enhanced expression of *Fbp1* and *Fbp2*. GAPDH activity was upregulated in IGT islets, but downregulated in both mild-HG and severe-HG islets and in INS-1 cells exposed to intermittent hyperglycaemia. We have shown previously that pharmacological inhibition of GAPDH recapitulates many of the metabolic effects seen with chronic hyperglycaemia and decreases insulin content in INS-1 cells^9^.

The limiting factor for GAPDH activity in the β-cell is probably the supply of the co-factor NAD^+^, as has been shown in neurons^50^. The ability of NAD^+^ supplementation to enhance OCR in severe-HG islets supports this view. This suggests that cytosolic supply of NAD^+^ is reduced in mild-HG and severe-HG islets (but not IGT islets). Studies on human islets have reported that the activity of the mitochondrial glycerol-3-phosphate dehydrogenase shuttle, the main source of cytosolic NAD^+^ in the β-cell, is impaired in T2D^51^. Additionally, chronic hyperglycaemia leads to an upregulation of the polyol pathway, which consumes cytosolic NAD^+^^9^. Taken together, these cellular processes will act to reduce the availability of cytosolic NAD^+^, thereby reducing GAPDH activity.

There was a progressive change in the pattern of metabolic gene expression from IGT to mild to severe diabetes. Interestingly, an increase in fasting glucose of just a few mM caused almost as many gene changes as severe diabetes, whereas the number of genes affected and the magnitude of the changes was less for IGT islets.

Both RNAseq and qPCR analysis revealed that certain metabolic genes were very sensitive to hyperglycaemia, their expression being altered in IGT islets and/or in INS-1 cells cultured at 8 mM glucose. This included the glycolytic genes, *Pfkl, Pfkfb2, Pfkfb3, Aldob* and *Pdk1*; the gluconeogenic genes, *Fbp1* and *Fbp2*; and the mitochondrial metabolism genes *Pdk1*, *Fh1* and *Cox6a2*. The expression of peroxisome proliferator-activated receptor gamma coactivator 1-alpha (Pgc-1α), which regulates mitochondrial biogenesis, was down-regulated in the qPCR (but not RNAseq) data. We consider *Pdk1* to be of special significance because it phosphorylates and inhibits pyruvate dehydrogenase and thereby reduces entry into the TCA cycle. It is likely that its enhanced expression plays a major role in impairing β-cell metabolism by causing the pooling of upstream metabolites that mediate changes in gene expression.

Although *Aldob* was significantly upregulated, no change in overall aldolase activity was observed, in either IGT or mild-HG islets. This may, in part, be because *Aldoa* is expressed at much higher levels in control islets than *Aldob* and may mask the increase in *Aldob*. Importantly, *Aldob* favours gluconeogenic activity^52^ and thus may contribute to the increase in glycogen in diabetic islets. *ALDOB* is upregulated in human T2D islets and negatively correlates with insulin secretion^14,53,54^.

We observed a reduction in *Trpm5* expression in both mild-HG and severe-HG islets. Knockout of *Trpm5* in mice abolishes GLP-1 stimulation of insulin release^55^. Thus, its reduced expression in diabetes, together with the observed down-regulation of *Gipr* may contribute to the impaired incretin response in IGT^56^ and T2D^57^.

It is worth noting that several genes that show very large relative increases in gene expression are expressed at very low levels in control islets. Consequently, small differences in basal transcript level may lead to marked variability in changes in relative expression, reducing significance levels. It may also mean that there will be little effect on functional protein activity in diabetes, due to low transcript levels, despite a marked change in gene expression.

Our results compare favourably with what is observed for islets isolated from living human patients exhibiting different levels of glycaemia. These show that *ALDOB* and genes related to mitochondrial oxidative phosphorylation are progressively perturbed from IGT to T2D^54^. In particular, there was a strong correlation between *ALDOB* and HbA1c.

The presence of glycogen is an indicator of impaired β-cell metabolism as it is not normally produced in β-cells, probably to avoid undesirable insulin release when blood glucose levels fall. Insufficient material was present to measure glycogen content in islets. However, chronic glucose elevation caused a progressive increase in glycogen in INS-1 cells that manifested at glucose concentrations as low as 8 mM. This is likely due to an increase in *Ppp1r3c* and the gluconeogenic enzymes *Aldob*, *Fbp1* and *Fbp2*, coupled with an inhibition of GAPDH and mitochondrial metabolism (indicated by reduced glucose-stimulated ATP-linked oxygen consumption) that will lead to pooling of upstream metabolites. The marked reduction of *G6pc2* seen in both mild and severe diabetic islets will also favour a rise in glucose-6-phosphate (the substrate for the glycogen synthesis pathway).

We observed that intermittent increases in glycaemia predisposed to diabetes development and progression both in vitro and in vivo. We therefore speculate that an increase in post-prandial glucose may be a factor driving development of IGT and its progression to diabetes. The higher the post-prandial glucose excursion and the longer it lasts, the more metabolic gene expression will change, impairing metabolism and insulin secretion. During the interval between meals, blood glucose will gradually return to normal levels, but gene expression may not do so fully. This will result in accumulating changes in gene expression that predispose to impaired β-cell metabolism. Consequently, there will be a vicious spiral in which impaired β-cell metabolism reduces insulin secretion, leading to elevated glucose and further adverse gene changes. In this way, IGT drives diabetes progression.

It is clear from our results that changes in gene expression and metabolism are already present in IGT islets. For example, they show changes in metabolic gene expression, enzyme activity and mitochondrial metabolism, as do INS-1 cells exposed to intermittent hyperglycaemia, or cultured for 48 h at 8 mM glucose. We argue that individuals with prolonged post-prandial glucose excursions will be predisposed to develop IGT. Such a predisposition may arise for a myriad of reasons, such as reduced β-cell capacity, genetic variants that lead to mild differences in metabolism, or stressors such as age (which reduces mitochondrial capacity) and obesity (that predisposes to insulin resistance).

At the cellular level, we postulate a key driver of diabetes progression is elevated NADH. The increase in NADH seen in control islets in response to acute glucose elevation is thought to reflect an increase in mitochondrial NADH. In severe-HG islets, NADH is elevated even at 2 mM glucose^9^. Such an increase argues that flux through the electron transport chain is saturated, leading to a build-up of NADH and FADH2 in the mitochondria. If this is large and prolonged, the mitochondrial glycerol phosphate shuttle is likely to become impaired, leading to a reduction in cytosolic NAD^+^ and elevation of cytosolic NADH. This will inhibit GAPDH activity, reducing later steps in glycolysis and leading to accumulation of upstream glycolytic metabolites. We have shown elsewhere that accumulation of upper glycolytic metabolites (such as fructose 1,6-bisphosphate, dihydroxyacetone phosphate, and glyceraldehyde-3-phosphate) leads to changes in gene expression that impair mitochondrial metabolism in β-cells^9^. Encouragingly, our results suggest that, at least in the short term, these changes in gene expression can be largely reversed by restoration of euglycaemia.

In conclusion, our results show that changes in glucose metabolism and metabolic gene expression occur in IGT mice, before the onset of sustained hyperglycaemia, and are observed in INS-1 cells subject to intermittent hyperglycaemia. A similar mechanism may underlie the loss of β-cell function during prediabetes in humans. Therapies that target glucose intolerance or very small changes in fasting glucose may therefore help prevent the seemingly inexorable decline in β−cell function seen in patients with T2D. Furthermore, early intensive glycaemic control of T2D is known to confer a near life-long reduction in risk of death or myocardial infarction^58^. However, rather than treating glucose intolerance with insulin or sulphonylureas, which can trigger hypoglycaemic side effects, we suggest a novel approach may be to prevent the accumulation of upper glycolytic metabolites that drive changes in gene expression—for example by partial reduction of upper glycolysis^28,59^. Better routine diagnostics for glucose intolerance (such as an oral glucose tolerance test or continuous glucose monitoring) are also needed as this is not detected by screening methods that measure only fasting or 2 h glucose.

## Methods

### Animal experiments

All animal studies were conducted in accordance with the UK Animals (Scientific Procedures) Act (1986) and approved by the local Department of Physiology Anatomy and Genetics (University of Oxford) ethical review committee. βV59M mice (which hemizygously express the inducible *Kir6.2-V59M* transgene specifically in their β-cells) were generated using a Cre-lox approach^39^. The background strain was C57BL6/J (Jackson Labs, USA). Both male and female mice were used. Transgene expression was induced at 12 weeks of age by subcutaneous injection of tamoxifen (Sigma) in corn oil: for severe diabetes, 10 µl/g body weight of 20 mg/ml; for mild diabetes 10 µl/g body weight of 2.5 mg/ml; for IGT mice, 10 µl/g body weight of 1.25 mg/ml. Paired control mice were injected with the same amount of tamoxifen. Tamoxifen-injected wild-type, RIPII-Cre-ER, and floxed Kir6.2-V59M littermates (pooled) were used as controls. All mice were bred in house from established stocks. Mice had unrestricted access to water and a regular chow diet (63% carbohydrate, 23% protein, 4% fat; Special Diet Services, RM3). They were maintained on a 12 h light-dark cycle at 21 °C and 45–55% relative humidity. Body weight and blood glucose levels were monitored routinely. Blood glucose levels were measured from the tail vein using the Freestyle Lite device and Freestyle Lite test strips (both Abbott). For studies of IGT, mild and severe diabetes, all mice were 14–15 weeks old when sacrificed.

For glibenclamide treatment, 2-week severely diabetic βV59M mice were implanted under general anaesthesia (2% isoflurane, Animal Care Ltd.) with slow-release glibenclamide pellets (Innovative Research of America) and sacrificed 2 weeks later when blood glucose levels had normalized ( < 10 mM)^29^. Tamoxifen-injected wild-type, RIPII-Cre-ER and floxed Kir6.2-V59M littermates (pooled) were used as controls. For studies of diabetes reversal, control and diabetic mice were 14 weeks old when sacrificed and glibenclamide-treated mice were 16 weeks old.

### Islet isolation and culture

Mice were killed by cervical dislocation and islets isolated essentially as described^9^. In brief, pancreata were inflated with collagenase solution at 1 mg/ml (Collagenase NB 8 Broad Range, Nordmark, S1745602), resected and placed in a water bath at 37 °C for 10 min. After washing, islets were purified on a Histopaque-1119 (Merck, #11191) / Histopaque-1083 (Merck, #10831) gradient. Islets were cultured overnight for respirometry, insulin secretion and enzyme studies but were lysed ~3–4 h after isolation for qPCR and RNAseq analysis. Islets were cultured in RPMI 1640 medium plus fetal bovine serum (10% (v/v), penicillin (100U/ml) and streptomycin (0.1 mg/ml) solution (all Thermo-Fischer-Scientific) at 37 °C, in a humidified atmosphere of 5%CO_2_/95% air. The glucose concentration of the media was altered to reflect the average fed blood glucose for each group: 11 mM glucose (control and IGT), 13 mM (mild-HG) or 25 mM glucose (severe-HG).

### INS-1 (832/13) cell culture

INS-1 (832/13) cells (abbreviated here as INS-1 cells) were originally developed by Claes Wollheim (Geneva). They were cultured in RPMI-1640 medium supplemented with 10% FBS, 1% Pen/Strep, 50 µM β-mercapto-ethanol, 1 mM Na-pyruvate, 10 mM HEPES, and 2 mM GlutaMAX (standard culture medium; all Sigma-Aldrich) in a humidified atmosphere of 5% CO_2_/95% air at 37 °C. INS-1 cells were maintained at 11 mM glucose and then cultured at different glucose (G) concentrations for 48 h to simulate euglycaemia (5 mM glucose), severe diabetes (16 and 25 mM glucose) and mild diabetes (8 and 11 mM). We term these cells 5G-, 8G-, 11G-, 16G- and 25G-cells. Additionally, we examined the impact of intermittent hyperglycaemia (IHG) by exposing INS-1 cells to 25 mM glucose for 4 separate 3 h periods over the course of 48 h, with cells being returned to 5 mM glucose during the interim periods.

### Insulin secretion

*INS-1 cells:* Cells were cultured at various glucose concentrations, as stated above, for 48 hr. On the day of the assay, cells were washed twice in Krebs-Ringer-bicarbonate buffer containing (in mM): 140 NaCl, 3.6 KCl, 0.5 NaH_2_PO_4_, 2 NaHCO_3_ (saturated with CO_2_), 1.5 CaCl_2_, 0.5 MgSO_4_, 10 HEPES (pH 7.4) and 0.1% (w/v) fatty acid free (FAF) BSA. Cells were pre-stimulated with 2 mM glucose Krebs buffer at 37 °C for 60 min, after which the buffer was removed, and cells were incubated with Krebs buffer containing 2 or 20 mM glucose for 30 min. The supernatant was collected and cells harvested either in acid ethanol (for total insulin content) or in RIPA lysis buffer (for protein content). Insulin levels in the supernatant and cell lysates were determined by insulin ELISA (Mercodia, Uppsala, Sweden). Insulin secretion and insulin content were normalised to protein content of the well.

*Islets*: Insulin secretion was assayed in triplicate on size-matched islets, isolated from control and diabetic β-V59M mice. Islets were pre-stimulated for 1 h in Krebs–Ringer bicarbonate buffer + 0.1% (w/v) FAF-BSA containing 2 mM glucose, and then stimulated for 1 h (10 islets/well) at 37 °C in Krebs–Ringer solution with either 2 mM or 20 mM glucose. Islets were harvested in acid ethanol to determine insulin content. Secreted and total insulin content were quantified by ELISA (Mercodia, Uppsala, Sweden). Data were expressed per islet per hour.

### Respirometry

The Seahorse XFe24 Extracellular Flux Analyser (Seahorse Bioscience, Copenhagen, Denmark) was used to assess a range of metabolic parameters by real-time monitoring of cellular oxygen consumption rate (OCR), as described by Haythorne et al (2019)^7^. INS-1 cells were cultured at various glucose concentrations, as stated above, for 48 h and washed in serum-free unbuffered RPMI medium (Agilent) containing 2 mM glucose for 1 h prior to measurement. Isolated islets were incubated overnight in RPMI supplemented with either 11 mM glucose (control islets), 25 mM glucose (severe-HG islets), 13 mM glucose (mild-HG islets) or 11 mM glucose (IGT islets). Islets were seeded at 50 islets/well in XF 24-well islet capture microplates in unbuffered RPMI containing 2 mM glucose and 0.1% FAF-BSA for 1–2 h prior to measurement.

Glucose-stimulated respiration was measured by addition of 20 mM glucose. Mitochondrial efficiency was assessed using compounds that inhibit specific mitochondrial processes: ATP-linked respiration (oligomycin) and proton leak (antimycin A + rotenone). Data are presented as either pmol O_2_/min/µg protein, pmol O_2_/min/50 islets or were normalised to the last baseline measurement prior to the addition of 20 mM glucose (100%). The % change in OCR following the addition of a compound/substrate was also calculated.

### SDS-PAGE and Western blotting

INS-1 (832/13) cells were cultured in RPMI medium at the indicated glucose concentration for 48 h. They were then serum starved for 1 h in HEPES-buffered saline containing (mM) 135 NaCl, 5 KCl, 1 MgCl_2_, 1 CaCl_2_, 10 HEPES (pH 7.4 with NaOH), plus 5, 8, 11, 16 or 25 mM glucose, followed by stimulation with 2 or 20 mM glucose (in saline) for 30 min. Cells were harvested in ice-cold RIPA buffer (Sigma-Aldrich) containing phosphatase inhibitors (Roche) and protease inhibitors (Roche).

Protein concentration was determined using a Pierce BCA protein assay kit (Thermo Fisher Scientific). Protein isolation and immunoblotting procedures were as described previously^60^. Briefly, 10 µg of protein lysates were subjected to SDS-PAGE and electrotransferred to nitrocellulose membrane, and immunoreactive proteins were identified by chemiluminescence. Primary and secondary antibodies used are listed in Supplementary Table 1. Gel bands were quantified by densitometry using Image Studio Lite software (Licor). mTORC1 signalling was determined by the ratio of phosphorylated (p-) and total ribosomal protein S6 (S6). AMPK signalling was determined by the ratio of phosphorylated (p-) and total AMPK protein.

Uncropped blots are available in the Supplementary information (Supplementary Fig. 6).

### RNA sequencing (control, IGT, mild, severely diabetic mice)

Islets were isolated from individual mice and total RNA isolated using the ReliaPrep™ RNA Cell Miniprep System (Promega) (Promega), according to the manufacturer’s instructions. RNA concentration and purity were initially determined using a NanoDrop ND-1000 spectrophotometer (Thermo Scientific). Library construction was performed at the Genomics and Bioinformatics Core at the Institute of Metabolic Science as described in ref.^61^. In brief, RNA quality was validated using Agilent Bioanalyzer 2100. All samples had RIN values between 7.4 and 10. 500 ng of total RNA was used for library construction using Illumina’s TruSeq Stranded mRNA prep kit following the manufacturer’s instructions. Indexed libraries were purified and validated using Agilent Bioanalyzer 2100. Sequencing libraries were pooled at equal molar concentration, and paired-end sequenced on an Illumina NovaSeq 6000 instrument (PE50) at the Genomics Core Facility, Cancer Research UK Cambridge Institute (Cambridge, UK). All samples were run on the sequencer at the same time.

Quality and adaptor trimming of sequenced transcripts was performed using cutadapt (v3.4). STAR (v2.7.3a) was used to align transcripts to the mouse genome (GRCm38). Raw counts were generated using featureCounts (v2.0.3). Quality control was performed using FastQC (v0.11.9). Differential gene expression analysis was performed in RStudio using DESeq2 (v1.42.0). Gene annotation was obtained from the Ensembl dataset held in BiomaRt (v2.58.2), combined with gene ontology annotation from goseq (v1.54.0). Receptor and ion channel lists were collated from the International Union of Basic and Clinical Pharmacology (IUPHAR) “targets and families” list. Gene expression is presented in transcripts per million. Differential expression was calculated using the Wald test (default in DESeq2), comparing IGT, mild-HG, or severe-HG to their respective negative controls. KEGG (Kyoto Encyclopedia of Genes and Genomes) analysis was performed using the KEGGREST package (v1.42.0). Metabolic genes (used for the PCA plot in Fig. 5a) were defined as genes involved in glycolysis, mitochondrial metabolism and the pentose phosphate pathway. Statistical analysis in Fig. 5c was performed by two-way ANOVA, including diabetes severity as a categorical variable, and *p*-values generated using Tukey’s HSD post-hoc test. RNA sequencing data have been deposited as described in the Data Availability statement below.

### RNA sequencing (control, severe diabetes, reversal mice)

Islets were isolated from control mice (*n* = 4), 2-week severely diabetic βV59M mice (n = 3) and 2-week severely diabetic mice (*n* = 4) treated with glibenclamide for a further 2 weeks to normalize blood glucose levels. RNA sequencing and analysis were performed as described in reference^7^. All samples were run at the same time, but while the data for control and severely diabetic mice have been reported previously^7^ that for reversal mice has not been reported before. RNA sequencing data have been deposited as described in the Data Availability statement below.

### qPCR

Total RNA was isolated using the ReliaPrep™ RNA Cell Miniprep System (Promega), according to the manufacturer’s instructions. RNA concentration was determined using a NanoDrop ND-1000 spectrophotometer (Thermo Scientific) and RNA reverse transcribed using Applied BiosystemsTM High-Capacity cDNA Transcription Kit (ThermoFisher). Quantitative PCR was performed using TaqMan probes (Supplementary Table 2) and the Applied Biosystems StepOne Plus Real-Time PCR system (Applied Biosystems). All reactions were performed in duplicate. Data were quantified according to the delta-delta Ct method with normalisation to the housekeeping gene*s Actb* (islets) or *Hspa8* and *Hprt1* (INS-1 cells).

### Glycogen measurements

INS-1 cells were cultured at the indicated glucose concentrations for 48 h. To help reduce background glucose contamination, cells were transferred to media containing 5 mM glucose for 30 min prior to sample collection. They were then washed twice with ice-cold PBS and lysed by sonication in ultrapure water. Lysates were immediately boiled for 10 min, centrifuged to pellet debris, and the glycogen content of the supernatant determined using the Glycogen Colorimetric/Fluorometric Assay Kit (Biovision K646) as per the manufacturer’s instructions. The protein concentration in the supernatant was determined by BCA assay.

### Enzyme activities

Enzyme activities were measured using commercial assay kits according to the manufacturer’s instructions: Hexokinase (Abcam, ab136957), Aldolase (Abcam, ab196994), Phospho-fructokinase (Abcam, ab155898), Glyceraldehyde 3-Phosphate Dehydrogenase (Abcam, ab204732) and Fructose-1,6-Bisphosphatase (Biovision, K590).

### Statistical analysis

Unless otherwise stated, results are presented as mean ± s.e.m. Unless otherwise stated, for islet experiments, n indicates the number of mice and for INS-1 cell studies, n indicates the number of biologically independent experiments. INS-1 cell experiments had 3 or more (usually 3) technical replicates and the mean value of all replicates was taken as *n* = 1 biologically independent experiment: statistical analysis was performed on biologically independent experiments. For oxygen consumption experiments, n indicates the number of replicates (wells).

Significance was tested using Student’s t test, one-way or two-way ANOVA as indicated in the figure legends, using Graphpad Prism software. Post-test corrections are indicated in the legends. Differences were considered statistically significant if *P*  <  0.05 using FDR corrections for multiple testing where applicable.

### Reporting summary

Further information on research design is available in the Nature Portfolio Reporting Summary linked to this article.

## Supplementary information

Supplementary Information

Description of Additional Supplementary Files

Supplementary Data 1

Supplementary Data 2

Supplementary Data 3

Supplementary Data 4

Supplementary Data 5

Reporting Summary

Transparent Peer Review file

## Source data

Source Data

## Data Availability

The authors declare that all data supporting the findings of this study are available within the paper, its Supplementary Information files, its Supplementary Data, or the Source Data file. Bulk RNA sequencing data for islets isolated from control, IGT, mild and severe diabetes mice are available in the EMBL-EBI ArrayExpress database (http://www.ebi.ac.uk/arrayexpress) under the accession number E-MTAB-16104. Bulk RNA sequencing data for islets from control and severe diabetes mice in Fig. 10 and supplementary Fig. 5 were previously published^7^ and are available in the European Nucleotide Archive (https://www.ebi.ac.uk/ena) under the accession number ERP114395. Reversal data, which were collected at the same time, are now also available under the accession number PRJEB105074. Mice containing the floxed Kir6.2/V59M transgene have been archived with the Mary Lyon Centre, MRC Harwell, UK. Supplementary Information accompanies this paper. Source data are provided with this paper.
