# Supplementary material for: Impaired glucose tolerance and mild diabetes induce β-cell dysfunction in mice
Source: Nat Commun. 2026 Apr 30;17:5921. doi: 10.1038/s41467-026-71528-3 (PMC13338056; doi:10.1038/s41467-026-71528-3)
Supplement: Supplementary file 1 — Supplementary Information [file 41467_2026_71528_MOESM1_ESM.pdf]

Supplementary information for:

Impaired glucose tolerance and mild diabetes induce β-cell dysfunction in mice

Authors: Elizabeth Haythorne[1,2*], Matthew Lloyd[1], Chris A. Smith[3], Martijn van de Bunt[4], Maria Rohm[1,5], Alice Elphick[1], Malgorzata Cyranka[1], Fiona M. Gribble[3], Frank Reimann[3], Frances M. Ashcroft[1*]

[1]Department of Physiology Anatomy and Genetics, Sherrington Building, Parks Road, Oxford, OX1 3PT, UK
[2]Institute for Neuroscience and Cardiovascular Research, University of Edinburgh, The Queen's Medical Research Institute, Edinburgh BioQuarter, Edinburgh, EH16 4TJ, UK
[3]Institute of Metabolic Science, University of Cambridge, Addenbrooke's Hospital, Cambridge, UK.
[4]Cytoki Pharma, Copenhagen, Denmark
[5] Institute for Diabetes and Cancer, Helmholtz Munich, Munich, Germany

*Joint corresponding authors

Elizabeth Haythorne: ehaythor@ed.ac.uk
Matthew Lloyd: matthew.lloyd@pharm.ox.ac.uk
Christopher Smith: cas228@medschl.cam.ac.uk
Martijn van de Bunt: mvdb@cytokipharma.com
Maria Rohm: maria.rohm@helmholtz-munich.de
Alice Elphick: alice.elphick@ccc.ox.ac.uk
Malgorzata Cyranka: MJCX@novonordisk.com
Fiona Gribble: fmg23@cam.ac.uk
Frank Reimann: fr222@cam.ac.uk
Frances Ashcroft: frances.ashcroft@dpag.ox.ac.uk

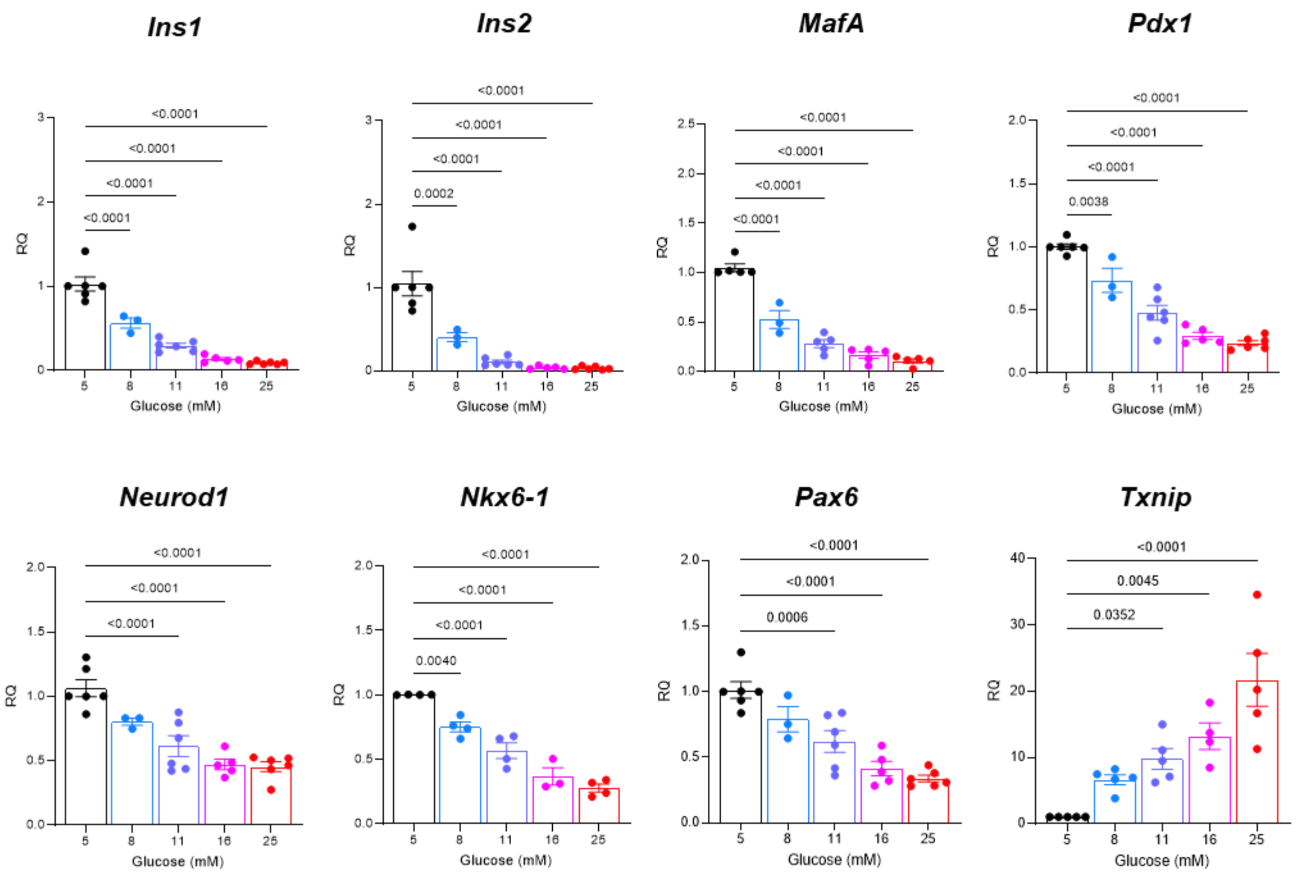

Supplementary Figure 1 ***Effects of chronic glucose on expression of selected genes in INS-1 cells***

mRNA levels for the indicated genes in INS-1 cells cultured for 48h at the indicated glucose concentrations (n=3-5). All panels show individual data points and mean ± s.e.m. N values indicate the number of biologically independent experiments. Two-tailed unpaired Student's t test. One-way ANOVA with Bonferroni post hoc test. Source data are provided as a Source Data file.

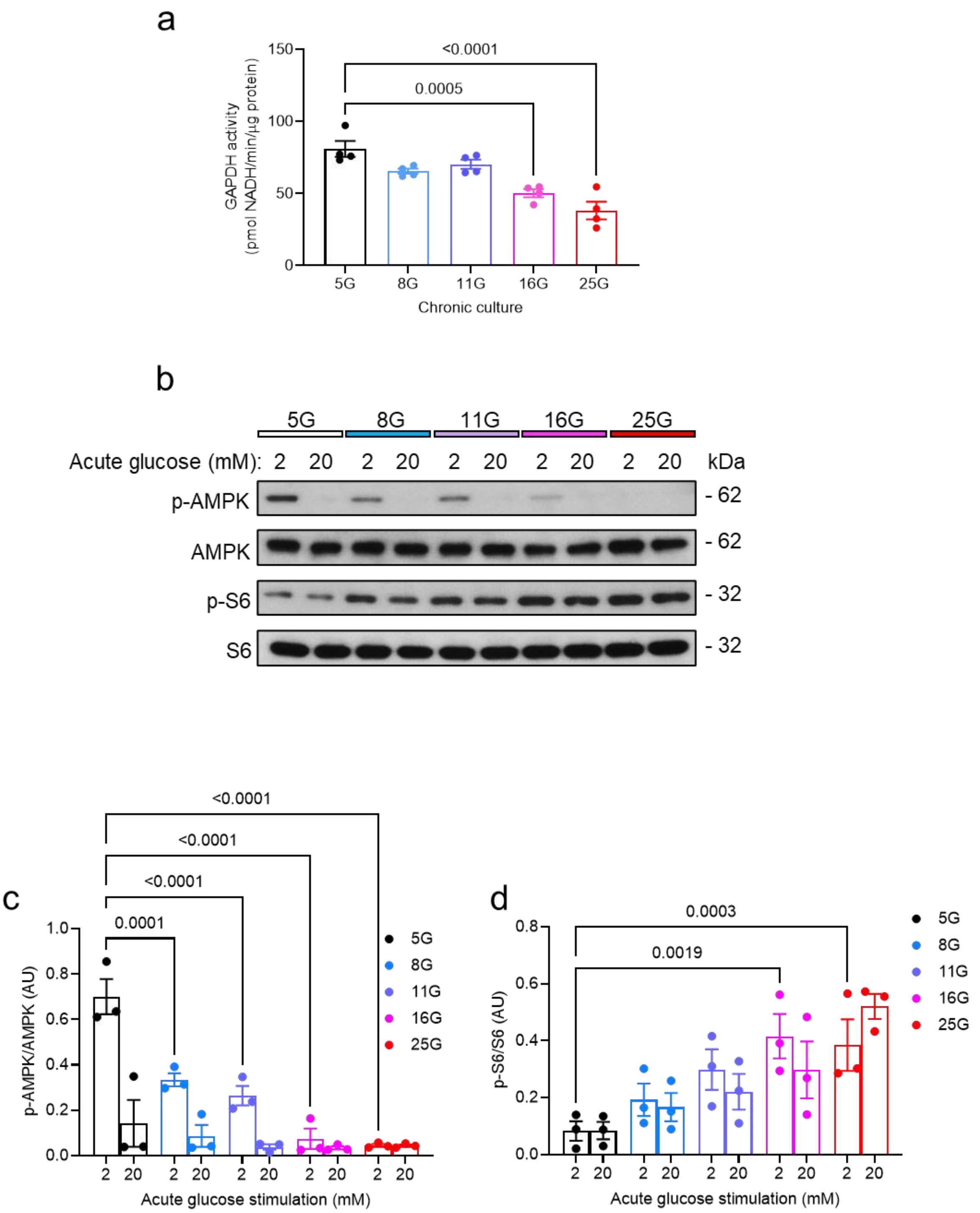

a
GAPDH activity (pmol NADH/min/µg protein)
<0.0001
0.0005
5G
8G
11G
16G
25G
Chronic culture
b
5G
8G
11G
16G
25G
Acute glucose (mM): 2 20 2 20 2 20 2 20 2 20
kDa
p-AMPK - 62
AMPK - 62
p-S6 - 32
S6 - 32
c
p-AMPK/AMPK (AU)
0.0001
<0.0001
<0.0001
<0.0001
Acute glucose stimulation (mM)
d
p-S6/S6 (AU)
0.0019
0.0003
Acute glucose stimulation (mM)

Supplementary Figure 2 ***Effects of chronic glucose on GAPDH and AMPK activity***

(a) Glyceraldehyde 3-phosphate dehydrogenase (GAPDH) activity in INS-1 cells cultured for 48h at the indicated glucose concentrations (n=4 biologically independent experiments).

(b) Representative Western blot of lysates from INS-1 cells cultured at the indicated glucose concentration (G) for 48h and then stimulated with 2mM or 20mM glucose for 30min. Phosphorylated (p) and total AMPK and S6.

(c,d) Quantitative densitometry analysis of p-AMPK/AMPK (c, n=3 experiments) and p-S6/S6 (d, n=3 experiments).

All panels (a,c,d) show individual data points and mean±s.e.m. N values indicate the number of biologically independent experiments. Two-way ANOVA with Bonferroni post hoc test (b,c) and one-way ANOVA with Bonferroni post hoc test (d). Source data are provided as a Source Data file.

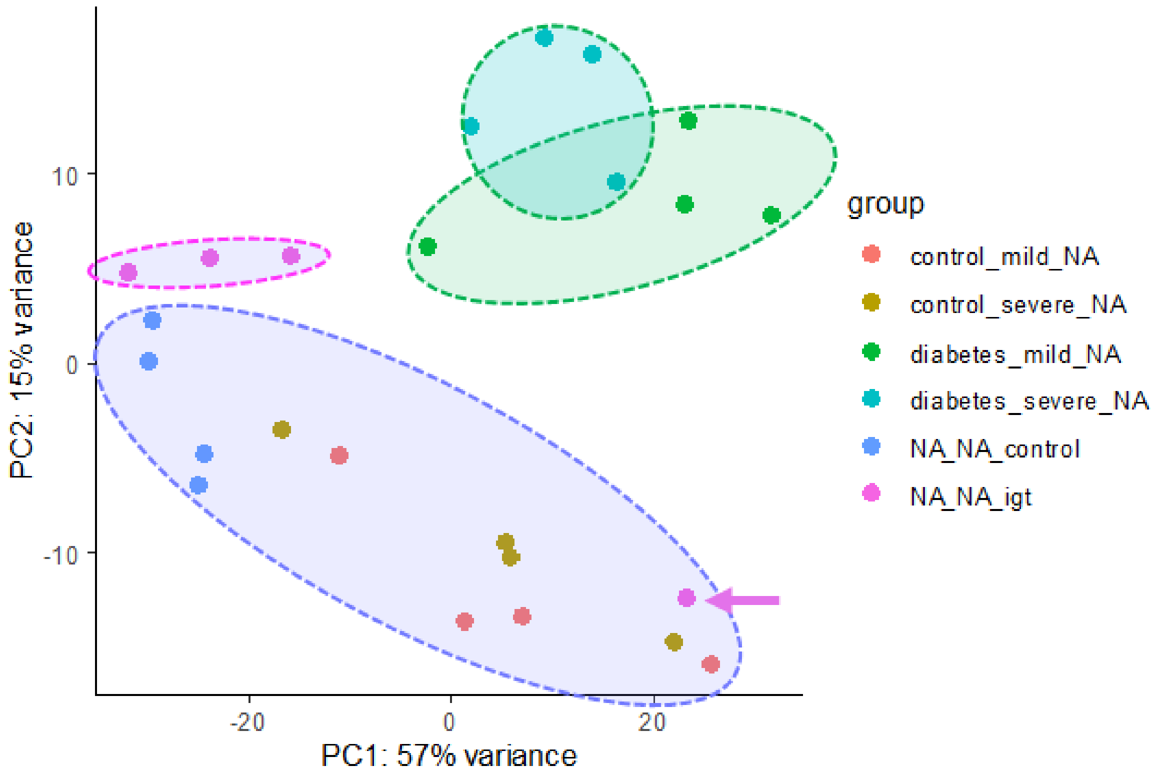

Supplementary Figure 3 ***PCA plot of RNAseq data from IGT, mild-HG and severe-HG mouse islets***

PCA plot of RNAseq data from IGT, mild-HG and severe-HG mouse islets and their respective controls. The plot is based on all genes identified. Control mice were injected with the same concentration of tamoxifen as their respective experimental cohorts. Arrow indicates a mouse that deviated from the others in the IGT set.

KEGG analysis

pathway	severe	mild	IGT
Citrate cycle (TCA cycle)	3.70E-06	3.10E-04	8.19E-02
Collecting duct acid secretion	9.66E-05	3.99E-04	9.59E-02
Lysosome	3.51E-04	1.14E-07	8.66E-03
Glycolysis / Gluconeogenesis	9.19E-04	1.70E-03	2.83E-02
Fatty acid degradation	1.35E-03	5.41E-01	4.15E-01
Central carbon metabolism in cancer	1.35E-03	1.24E-03	2.30E-02
Pentose phosphate pathway	1.42E-03	1.93E-02	8.56E-03
Valine, leucine and isoleucine degradation	1.88E-03	7.97E-03	3.40E-02
Fructose and mannose metabolism	2.03E-03	2.78E-06	1.55E-03
MAPK signaling pathway	2.08E-03	2.20E-08	1.24E-01
Endocytosis	2.27E-03	2.05E-01	7.43E-01
Butanoate metabolism	2.49E-03	6.14E-02	1.84E-01
Oxidative phosphorylation	2.64E-03	1.14E-02	7.38E-01
Alanine, aspartate and glutamate metabolism	3.48E-03	3.06E-01	1.37E-01
HIF-1 signaling pathway	5.86E-03	2.84E-05	1.54E-01
mTOR signaling pathway	6.60E-03	3.34E-02	7.27E-01
Pyruvate metabolism	6.83E-03	3.55E-02	8.56E-02
Colorectal cancer	6.97E-03	5.31E-02	5.26E-01
Gastric cancer	8.01E-03	1.56E-04	1.54E-01
Insulin signaling pathway	8.15E-03	4.94E-03	1.60E-01

**total no. of significant pathways**	216	240	46

Supplementary Figure 4 ***Pathway analysis of RNAseq data***

KEGG analysis of RNAseq data showing the top 20 pathways affected in severe-HG islets (listed in order of significance). The significance of these pathways in mild-HG and IGT islets is included for comparison. Pathways in which p<0.05 are highlighted in yellow. The total number of significant pathways was 216 in severe-HG islets, 240 in mild-HG islets and 46 in IGT islets.

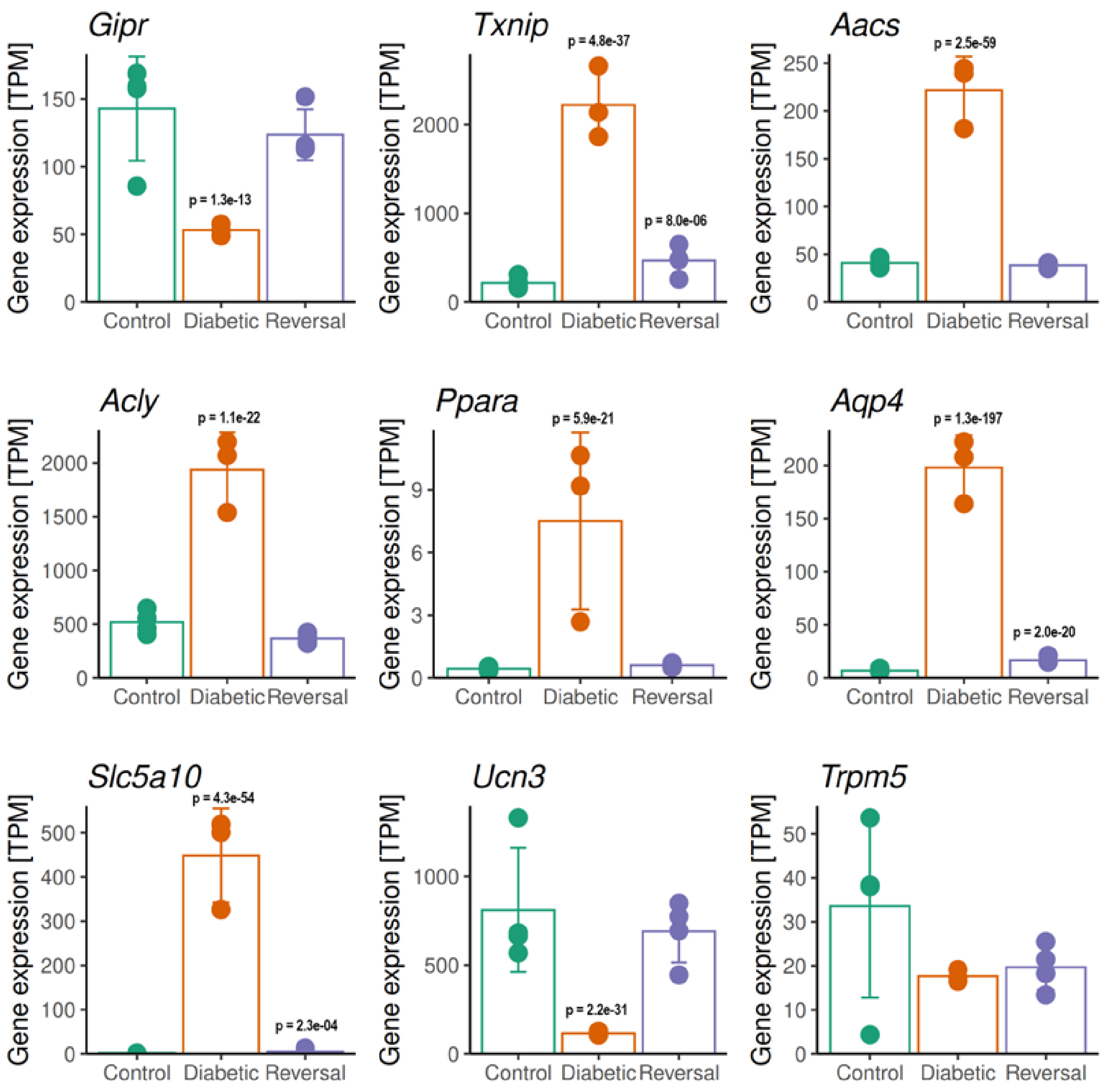

Supplementary Figure 5 ***Hyperglycaemia-induced changes in gene expression are reversed following restoration of euglycaemia***

Expression of indicated genes in islets isolated from control mice (green, n=4), 2-wk severe-HG mice (diabetic, red, n=3) and 2-wk severe-HG mice subsequently treated for 2-wk with glibenclamide to restore euglycaemia (reversal, blue, n=4). Genes that show major changes in diabetes have been selected. Expression is shown as transcripts per million (TPM). Mean (±SD) and individual data points are shown. Significance against control data is indicated. N values indicate the number of biologically independent experiments. Exact test for a difference in mean between two groups of negative binomial random variables as implemented in the R package *edgeR* with multiple testing correction using the Benjamini–Hochberg procedure. Source data are provided as a Source Data file.

Uncropped blots corresponding to Supplementary Fig.2b

mTORC1 – S6, pS6

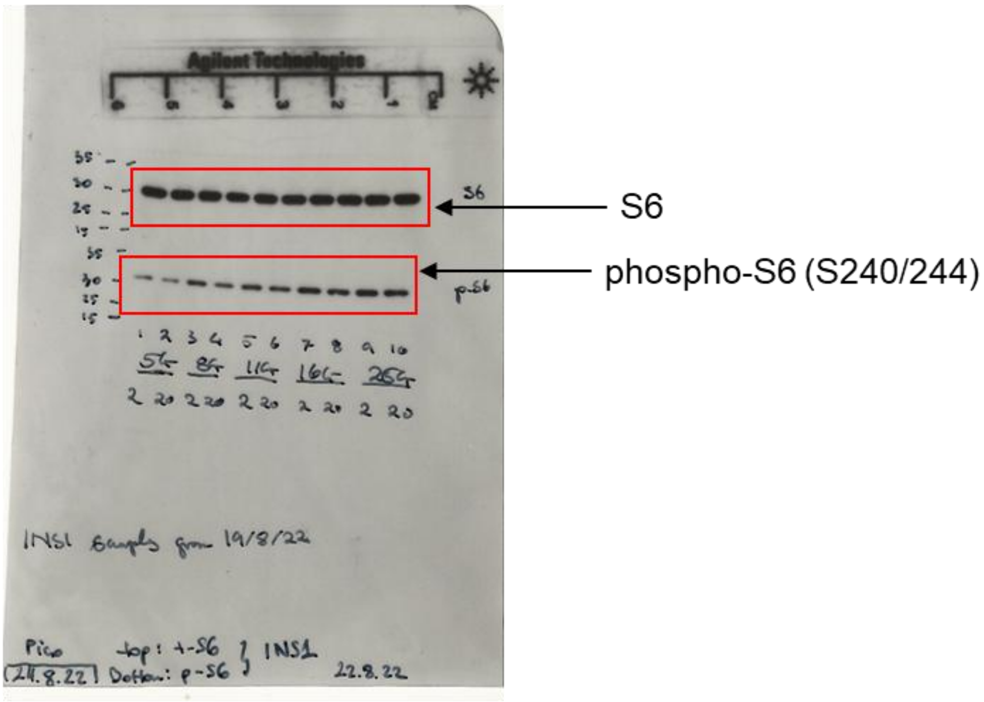

AMPK – AMPK, p-AMPK

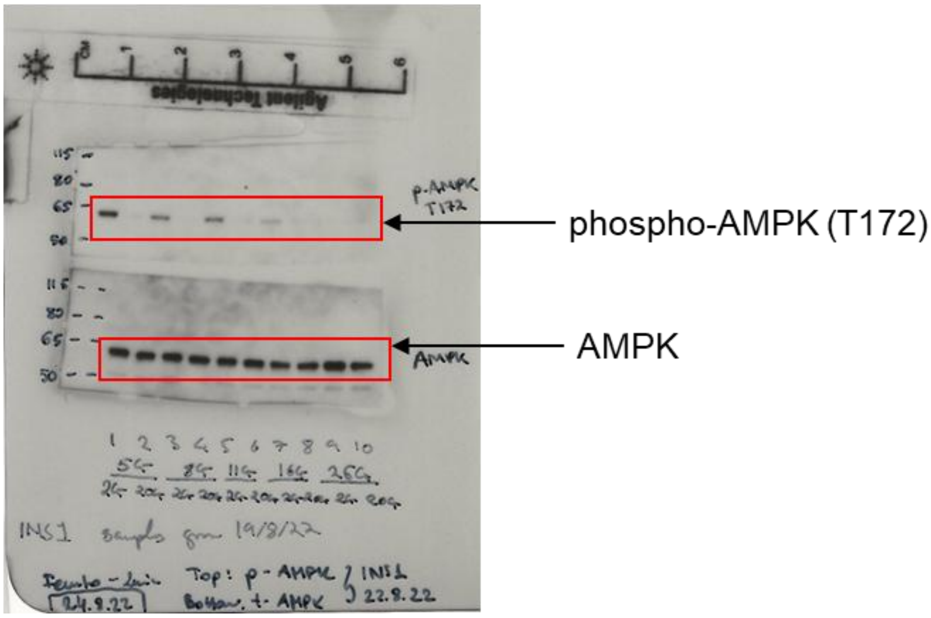

Supplementary Figure 6 ***Original Western blots for the data shown in Supplementary Figure 2b***

(above) Uncropped original Western blot of lysates from INS-1 cells cultured at the indicated glucose concentration (G) for 48h and then stimulated with 2mM or 20mM glucose for 30min. Total S6 and phosphorylated S6 (at S240 and 244) are indicated. The red boxes indicate the parts of the blot shown in Supplementary Figure 2b.

(below) Uncropped original Western blot of lysates from INS-1 cells cultured at the indicated glucose concentration (G) for 48h and then stimulated with 2mM or 20mM glucose for 30min. Total AMPK and phosphorylated AMPK (at T172) are indicated. The red boxes indicate the parts of the blot shown in Supplementary Figure 2b.

Western blot antibodies:

Antibody	Source	Dilution
Rabbit anti-phospho-AMPKα-T172 antibody	Cell Signaling Technology, cat. #2535	1:1000
Rabbit anti-AMPKα antibody	Cell Signaling Technology, cat. #2532	1:1000
Rabbit anti-phospho-S6-S240/244 antibody	Cell Signaling Technology, cat. #5364	1:4000
Rabbit anti-S6 antibody	Cell Signaling Technology, cat. #2217	1:4000

Supplementary Table1

List of antibodies used

Gene	Catalogue number
Actb	Mm02619580_g1
Aldob<br>*Cox6a2*	Mm00523293_m1 (mouse) and Rn01768292_m1 (rat)<br>Mm00438295_g1
Eno1	Mm01619597_g1
Fbp1<br>*Fbp2*	Mm00490181_m1<br>Mm00484280_m1
Fh1	Mm01321349_m1
Gapdh	Rn01775763_g1
G6pc2	Mm00491176_m1
Hspa8	Rn00821191_g1
Hprt1	Rn01527840_m1
Idh2	Rn01478119_m1
Ins1<br>*Ins2*<br>*Mafa*	Rn02121433_g1<br>Mm00731595_gH<br>Rn00845206_s1
Mdh2	Mm00725890_s1
Ndufa4	Mm00809672_s1
Ndufs2	Rn01411711_m1
Ndufs8	Mm00523063_m1
Neurod1	Rn01280117_m1
Nkx6-1	Rn01450076_m1
Ogdh<br>*Pax6*	Mm00803119_m1<br>Rn00689608_m1
Pdk1	Mm00554306_m1 (mouse) and Rn00587598_m1 (rat)
Pdx1	Rn00755591_m1
Pfkfb2	Mm00435575_m1
Pfkfb3	Mm00504650_m1 (mouse) and Rn00678825_m1 (rat)
Pfkl	Mm00435587_m1
Ppargc1a	Mm01208835_m1
Ppp1r3c	Mm01204084_m1
Sdha	Rn00590475_m1
Txnip	Mm00452393_m1

Supplementary Table 2

List of Taqman probes used
