# Supplementary material for: Impaired glucose tolerance and mild diabetes induce β-cell dysfunction in mice
Source: Nat Commun. 2026 Apr 30;17:5921. doi: 10.1038/s41467-026-71528-3 (PMC13338056; doi:10.1038/s41467-026-71528-3)
Supplement: Supplementary file 2 — Description of Additional Supplementary Files [file 41467_2026_71528_MOESM2_ESM.pdf]

SUPPLEMENTARY DATA

Supplementary Data 1

Differential gene expression in severe-HG mice and their respective controls. Changes in mRNA are given as log2-fold changes. Only significant changes in gene expression are listed.

Supplementary Data 2

Differential gene expression in mild-HG mice and their respective controls. Changes in mRNA are given as log2-fold changes. Only significant changes in gene expression are listed.

Supplementary Data 3

Differential gene expression in IGT mice and their respective controls. Changes in mRNA are given as log2-fold changes. Only significant changes in gene expression are listed.

Supplementary Data 4

Genes used for the PCA plot in Fig.5a.

Supplementary Data 5

Differential gene expression in control mice, 2-week diabetic mice and reversal mice (2wk diabetes followed by 2-wk of glibenclamide therapy). Changes in mRNA are given as log2-fold changes and significance indicated as FDR-corrected P-values. A FDR < 0.01 is considered significant.
