# Supplementary material for: Impaired glucose tolerance and mild diabetes induce β-cell dysfunction in mice
Source: Nat Commun. 2026 Apr 30;17:5921. doi: 10.1038/s41467-026-71528-3 (PMC13338056; doi:10.1038/s41467-026-71528-3)
Supplement: Supplementary file 9 — Transparent Peer Review file [file 41467_2026_71528_MOESM9_ESM.pdf]

nature portfolio

Peer Review File

Impaired glucose tolerance and mild diabetes induce β-cell dysfunction

Corresponding Author: Dr Elizabeth Haythorne

This file contains all reviewer reports in order by version, followed by all author rebuttals in order by version.
Parts of this Peer Review File have been redacted as indicated to maintain the confidentiality of unpublished data.

Version 1:

Reviewer comments:

Reviewer #1

(Remarks to the Author)
Haythorne and colleagues use INS1 cells and islets isolated from transgenic mice to study the impact of various degrees of (hyper)glycemia on metabolic parameters of pancreatic beta cells (or islets). Using immortalized rat INS1 cells, the authors show increasing glucose concentrations during 48 h culture decrease insulin content, GSIS, increase basal OCR, reduce 20G OCR when baseline-normalized, changed gene expression in glycolysis, mitochondrial metabolism and glycogen synthesis/stores. The authors also show that transient episodes of hyperglycemia in INS1 cells are enough to recapitulate some (but not all) of these metabolic effects. The authors then go in vivo in a K ATP channel opener transgenic mouse that allows to tam dose-dependently reduced insulin/increase glycemia and use isolated islets and RNAseq and qPCR to show that glycolytic and gluconeogenesis genes are upregulated while TCA-related genes are rather downregulated, even in the islets isolated from mice with weak hyperglycemia. They also show that some of the observed expression changes in glycolysis/gluconeogenesis metabolism translate to changes in enzyme activity.
The manuscript seems solid with good methods and sound results. It is somewhat incremental to work of others (and earlier publications of the same group). The highlight is that these (previously published phenotypes) already happen at a relatively small increase in glycemia.
I'm a bit sceptical about the use an immortalized cell line to study beta-cell metabolism. After all, INS1 cells is a cancer cell line that has relatively little to do with primary cells and has a profoundly different metabolism. I acknowledge that key findings are reproduced in primary mouse islets.
The authors data might suggest that already modest changes in glycemia in human disease (e.g prolonged weak hyperglycemia in prediabetes) might have strong influences on beta-cell function. I think the manuscript would profit from an additional in vivo experiment that tackles this more translational thinking. The authors could use their IGT model (or another mild diabetes model like an islet amyloidosis model) and then do an intervention study to show whether these changes are reversible. E.g. do the same 2 week mild hyperglycemia study, but then normalized glycemia with insulin therapy or SGLT2 inhibition to use qPCR or enzyme activities to check whether key findings are reversed. Such an experiment would increase the level of novelty.

Reviewer #2

(Remarks to the Author)
Summary:
Haythorne and colleagues aim to identify the impact of mild chronic elevations in glycemia on β-cell function. To do this, they utilized the INS-1 832/13 rat insulinoma cell line and islets isolated from their previously developed βV59M mouse model. Cell lines were stimulated with varying concentrations of glucose to simulate euglycemia, mild hyperglycemia, and diabetic conditions, and islets were isolated from mice treated with varying levels of tamoxifen that elicited different levels of glucose dyshomeostasis. Cell and islet function, mitochondrial function, and gene expression signatures were subsequently evaluated.

Overall Comments:
This study was well designed and executed and beautifully written. The findings in this study pursue a simple yet important question that is often overlooked in the field of diabetes research. They also pursue a comprehensive series of transcriptional, metabolic, physiologic, and enzymatic effects to generate a well-rounded picture of changes that occur in

beta cells subjected to elevations of blood glucose more likely seen in T2D rather than extreme glucotoxicity that is not likely to occur in humans. There are some limitations to the work that need to be clarified that are detailed below.

Major Comments:
1. A major concern is that while the authors nicely put forth a series of changes in mitochondrial metabolism and glycolysis through enzymatic and transcriptional effects that are consequential in beta cell decline to modest hyperglycemia, there is not an underlying mechanistic explanation for the root cause(s) of these changes by glucose. The authors posit roles for mitochondrial NADH and also observe interesting changes with GAPDH and mTOR signaling that could be pursued, but they did not do so. What leads to the transcriptional effects on the key genes observed? Unfortunately, these are also not assessed. Without deeper mechanistic insights into the causes of these glucose-induced effects, the studies could be considered descriptive. Could targeting one of these pathways remediate the phenotypes observed? This would substantially strengthen the observations in the paper.
2. The authors argue in the conclusion that a potential treatment option for those in the prediabetes stage is to “prevent the accumulation of upper glycolytic metabolites” – is there a rescue experiment that can be performed on the IGT mice to help bolster this argument?
3. Studies in the βV59M mouse model were lacking in some key validation studies. While they showed a nice dose-dependent effect of tamoxifen to increase glucose, the presumption would be that increases in glucose were related to recombination efficiency by their RIP2-CreER model at the ROSA locus. This would suggest that in their IGT group, very few beta cells would be recombined while in their severe HG group, many beta cells would be recombined. However, they did not demonstrate this.
4. As a corollary to this point, there is a presumption that results in the IGT group are due to modest hyperglycemia. An alternative explanation could be that all of their studies were done in whole islets and the mechanistic effects in the recombined beta cells in the IGT group were actually the same as the recombined beta cells in the severe HG group that were simply diluted out in whole islets mixed with beta cells that were never recombined. As studies were always performed in whole islets and single beta cells were not assessed, we do not know if the IGT islets simply had a few very poor functioning cells surrounded by normal cells. To answer the authors’ hypothesis, they would need to show that the non-recombined cells in the βV59M mouse model of the IGT group functioned similarly poorly as the βV59M expressing cells (i.e. the effects are only due to hyperglycemia and not the βV59M mutation per se).
5. Another study that would be convincing of their hypothesis in the βV59M mouse model would be to isolate islets from these IGT and mild HG models and demonstrate that their function and transcriptional profiles could be restored after removal from hyperglycemia.
6. While the data in this manuscript are convincing, there are obvious limitations to both systems in the translation to human diabetes. Some confirmatory studies using human models or data would be highly supportive. Indeed, this could be studies in human islets/Endo bh-3/5 cells or alternatively comparison of their gene expression results with data from human islet donors with pre-diabetes/IGT (see for example Wigger, et al. Nature Metabolism PMID 34183850 or Bandesh, et al. bioRxiv 2025 PMID 39896672).

Minor Comments:
1. Check for consistency in “INS-1 cells” (sometimes referred to in manuscript as INS1-cells)
2. Figure 1A – any significant changes in the low glucose conditions? Looks like trending down for each subsequent group
3. Figure 2A-D, line 160 – these genes are dramatically altered in 25G cells, but in 8G “dramatically” seems a bit of an overstatement for everything except perhaps Aldob. Also, Gapdh really doesn’t look dramatically altered in any group. Please consider tempering the wording here.
4. Line 261 – the “A” in Acaca is not italicized
5. Line 262, 264, 269, 275 – add figure references at the end of the sentences; these are the only ones the reviewer noticed, but please check the rest of the text to ensure proper figure citing throughout
6. Very minor comment – when listing genes for both Figures 5 and 6, listing the genes in the text in the order they appear in the figures would make it easier for the reader to cross-check between the two
7. Figure 7B-K – Data points are varying sizes in each of the graphs and would look a little cleaner if they were consistent sizes
8. Figure 7J also looks slightly vertically stretched compared to the other graphs in the figure
9. Figure 8B&F and 8D&H – Data points and axis titles are different sizes and would look cleaner if they were consistent with each other
10. Line 440 – change “beta cell” to “β-cell” for consistency

Version 2:

Reviewer comments:

Reviewer #1

(Remarks to the Author)
Thank you for taking my advice and adding the data of the in vivo intervention study. Even though not all readouts were normalized (as expected in complex biological systems) I think this adds a nice “translational” angle to the in vitro-heavy story.
One detail: Please make sure the number of animals is correctly outlined. New Figure 10 says 4,4,4 (legend) while the figure looks more like 4,3,4 (difficult to say). Suppl. Fig. 5 (derived from the same data I guess) states 4,3,4. If the number of samples are not the same, the authors should state why they excluded a dataset.

I have no further questions/suggestions.

Reviewer #2

(Remarks to the Author)
The authors adroitly addressed remaining concerns in the manuscript. I have no remaining concerns other than having the authors include the data for reviewer figure #1 which included studies in islet microspheres from Insphero as well as INS-1 cells with mannoheptulose into the manuscript. These data would likely fit nicely within Figure 10 of their manuscript and would add weight related to the inclusion of studies in human samples.

I commend the authors on an excellent study.

Open Access This Peer Review File is licensed under a Creative Commons Attribution 4.0 International License, which permits use, sharing, adaptation, distribution and reproduction in any medium or format, as long as you give appropriate credit to the original author(s) and the source, provide a link to the Creative Commons license, and indicate if changes were made.
In cases where reviewers are anonymous, credit should be given to 'Anonymous Referee' and the source.
The images or other third party material in this Peer Review File are included in the article's Creative Commons license, unless indicated otherwise in a credit line to the material. If material is not included in the article's Creative Commons license and your intended use is not permitted by statutory regulation or exceeds the permitted use, you will need to obtain permission directly from the copyright holder.
To view a copy of this license, visit https://creativecommons.org/licenses/by/4.0/

Reply to the reviewers

We are delighted that both reviewers found our paper to be of interest, praised the design, and execution of our experiments, and commented that the paper is well written and addresses an important but neglected topic. We thank them for their very helpful comments and criticisms which we address individually below.

Reviewer #1 (Remarks to the Author):
Haythorne and colleagues use INS1 cells and islets isolated from transgenic mice to study the impact of various degrees of (hyper)glycemia on metabolic parameters of pancreatic beta cells (or islets). Using immortalized rat INS1 cells, the authors show increasing glucose concentrations during 48 h culture decrease insulin content, GSIS, increase basal OCR, reduce 20G OCR when baseline-normalized, changed gene expression in glycolysis, mitochondrial metabolism and glycogen synthesis/stores. The authors also show that transient episodes of hyperglycemia in INS1 cells are enough to recapitulate some (but not all) of these metabolic effects. The authors then go in vivo in a K ATP channel opener transgenic mouse that allows to tam dose-dependently reduced insulin/increase glycemia and use isolated islets and RNAseq and qPCR to show that glycolytic and gluconeogenesis genes are upregulated while TCA-related genes are rather downregulated, even in the islets isolated from mice with weak hyperglycemia. They also show that some of the observed expression changes in glycolysis/gluconeogenesis metabolism translate to changes in enzyme activity.
The manuscript seems solid with good methods and sound results. It is somewhat incremental to work of others (and earlier publications of the same group). The highlight is that these (previously published phenotypes) already happen at a relatively small increase in glycemia.
I'm a bit sceptical about the use an immortalized cell line to study beta-cell metabolism. After all, INS1 cells is a cancer cell line that has relatively little to do with primary cells and has a profoundly different metabolism. I acknowledge that key findings are reproduced in primary mouse islets.
The authors data might suggest that already modest changes in glycemia in human disease (e.g prolonged weak hyperglycemia in prediabetes) might have strong influences on beta-cell function. I think the manuscript would profit from an additional in vivo experiment that tackles this more translational thinking. The authors could use their IGT model (or another mild diabetes model like an islet amyloidosis model) and then do an intervention study to show whether these changes are reversible. E.g. do the same 2 week mild hyperglycemia study, but then normalized glycemia with insulin therapy or SGLT2 inhibition to use qPCR or enzyme activities to check whether key findings are reversed. Such an experiment would increase the level of novelty.

We have addressed this question in two ways. First, we performed the suggested intervention study. We treated mice that had been severely diabetic for 2 weeks with the sulphonylurea glibenclamide, which rapidly normalised blood glucose levels (as described in Brereton et al, 2019). After 2 weeks of therapy, we sacrificed these mice, isolated their islets and carried out RNAseq. These data are now presented in a new Fig.10 and a new Supplementary Fig.5, and described in the text. They show that most genes have almost fully recovered within 2 weeks, after restoration of normal blood glucose levels.

In experiments that form part of a separate paper, we also showed that the effect of hyperglycaemia on insulin secretion from human islets or from INS-1 cells could be largely or partially reversed by subsequent partial inhibition of glucokinase activity with mannoheptulos e. Changes in gene expression and glycogen content in INS-1 cells were also revers ed. However, insulin content did not substantially recover on the time scale of our experiment s, which we consider may be due to the slow time course of reactivation of insulin ge ne expression on return to euglycaemia. We include a figure illustrating this for the revie wer (Reviewer Fig. 1a-f). Reviewer Figure 1 has been retracted, The data can be found in th

Reviewer #2 (Remarks to the Author):
Summary: Haythorne and colleagues aim to identify the impact of mild chronic elevations in glycemia on β-cell function. To do this, they utilized the INS-1 832/13 rat insulinoma cell line and islets isolated from their previously developed βV59M mouse model. Cell lines were

stimulated with varying concentrations of glucose to simulate euglycemia, mild hyperglycemia, and diabetic conditions, and islets were isolated from mice treated with varying levels of tamoxifen that elicited different levels of glucose dyshomeostasis. Cell and islet function, mitochondrial function, and gene expression signatures were subsequently evaluated.

Overall Comments: This study was well designed and executed and beautifully written. The findings in this study pursue a simple yet important question that is often overlooked in the field of diabetes research. They also pursue a comprehensive series of transcriptional, metabolic, physiologic, and enzymatic effects to generate a well-rounded picture of changes that occur in beta cells subjected to elevations of blood glucose more likely seen in T2D rather than extreme glucotoxicity that is not likely to occur in humans. There are some limitations to the work that need to be clarified that are detailed below.

Major Comments:
1. A major concern is that while the authors nicely put forth a series of changes in mitochondrial metabolism and glycolysis through enzymatic and transcriptional effects that are consequential in beta cell decline to modest hyperglycemia, there is not an underlying mechanistic explanation for the root cause(s) of these changes by glucose. The authors posit roles for mitochondrial NADH and also observe interesting changes with GAPDH and mTOR signaling that could be pursued, but they did not do so. What leads to the transcriptional effects on the key genes observed? Unfortunately, these are also not assessed. Without deeper mechanistic insights into the causes of these glucose-induced effects, the studies could be considered descriptive. Could targeting one of these pathways remediate the phenotypes observed? This would substantially strengthen the observations in the paper.

Using high glucose cultured cells and diabetic islets, we previously showed that partial (chronic) inhibition of GAPDH at low glucose causes accumulation of upstream metabolites and the same changes in gene expression as chronic hyperglycaemia (Haythorne et al 2022, reference 7). In addition, inhibition of glucokinase prevented the effects of hyperglycaemia. This suggests that an upper glycolytic metabolite, lying between glucose-6-phosphate and GAPDH, is implicated in causing the observed changes in gene expression and oxidative phosphorylation. We also showed that some of these changes induced by chronic hyperglycaemia are driven by activation of mTORC1 as they could be prevented by inhibition of S6 kinase (a downstream target of mTORC1). The data provided in this paper supports this idea and extends it to show that culture of INS-1 cells at as little as 8mM glucose is sufficient to initiate changes in GAPDH, mTORC1 and AMPK activity.

We now provide further evidence for the role of NAD^+ depletion in driving impaired mitochondrial metabolism in chronic hyperglycaemia. Specifically, we show that supplementation of the culture medium with NMN, which is known to increase cytosolic NAD^+, enhances GAPDH activity, restores basal oxygen consumption and partially restores glucose-stimulated OCR and ATP-dependent OCR in diabetic islets. It also partially restores glucose-stimulated insulin secretion, and causes a small increase in insulin content. The data is now presented in a New Fig.9. These effects are expected if limitation of NAD^+ is inhibiting GAPDH and causing impaired metabolism and pooling of upstream metabolites. NMN supplementation has previously been reported to improve glucose tolerance and insulin secretion in aged HFD-induced diabetic mice (Yoshino et al. Cell Metab 14, 528-36, 2011) and potentiate post-prandial insulin secretion in humans (Yamane T, et al., Clin Nutr ESPEN. 56:83-86, 2023)

2. The authors argue in the conclusion that a potential treatment option for those in the prediabetes stage is to “prevent the accumulation of upper glycolytic metabolites” – is there a rescue experiment that can be performed on the IGT mice to help bolster this argument?

We have shown previously that partial inhibition of glucokinase prevents the deleterious effects of chronic hyperglycaemia of the beta-cell (Haythorne et al. 2022, reference 7 in the paper). As discussed above we have also found that by reducing glucokinase activity back to normal levels, mannoheptulose can restore insulin secretion in hyperglycaemic INS-1 cells and in human islets. It can also reverse the effects of hyperglycaemia on gene expression in INS-1 cells (see reply to Reviewer 1 and Reviewer Figure 1). (Figure 1 has been redacted, see reply to Reviewer 1) Unfortunately, we cannot do this experiment in vivo as mannoheptulose is prohibitively expensive. However, patients with heterozygous loss-of-function mutations in glucokinase do not develop the secondary complications of diabetes, and although they have elevated blood glucose this does not worsen over time. This suggests that partial reduction of glucokinase activity (back to non-diabetic levels) might be a novel therapeutic strategy for diabetes. We have discussed this idea previously in detail in a short review (Trends in Endocrinology and Metabolism 34, p119) and also in reference 28. However, our aim in this paper was not to offer a novel therapy for diabetes but to demonstrate that even small changes in plasma glucose levels have a detrimental effect on β-cell function and underline the progressive decline in β-cell function. This means it is important to control blood

3. *Studies in the βV59M mouse model were lacking in some key validation studies. While they showed a nice dose-dependent effect of tamoxifen to increase glucose, the presumption would be that increases in glucose were related to recombination efficiency by their RIP2-CreER model at the ROSA locus. This would suggest that in their IGT group, very few beta cells would be recombined while in their severe HG group, many beta cells would be recombined. However, they did not demonstrate this.*

4. As a corollary to this point, there is a presumption that results in the IGT group are due to modest hyperglycemia. An alternative explanation could be that all of their studies were done in whole islets and the mechanistic effects in the recombined beta cells in the IGT group were actually the same as the recombined beta cells in the severe HG group that were simply diluted out in whole islets mixed with beta cells that were never recombined. As studies were always performed in whole islets and single beta cells were not assessed, we do not know if the IGT islets simply had a few very poor functioning cells surrounded by normal cells. To answer the authors' hypothesis, they would need to show that the non-recombined cells in the βV59M mouse model of the IGT group functioned similarly poorly as the βV59M expressing cells (i.e. the effects are only due to hyperglycemia and not the βV59M mutation per se).

Reply to comments 3 and 4. We agree with the reviewer that many cells will not be recombined in the IGT and mild diabetes groups. However, as they point out in comment 5, the critical issue is not how many beta-cells are recombined but whether the effect of elevated glucose (IGT, mild or severe) affects all beta-cells in a similar way, or if it is specific to those beta-cells in which the *KCNJ11* mutation is expressed - or if the effects observed are due to K_{ATP} channel activation and not to glucose. We have unpublished single cell RNAseq data that shows the same changes in metabolic gene expression (e.g. upregulation of *aldob, pdk1*) in β-cells that do not express the Kir6.2-V59M mutation as in those that do express the mutation. This indicates that the changes in gene expression are driven by glucose and not the βV59M mutation *per se*. We attach a confidential figure illustrating this point for the reviewer (Reviewer Fig. 2). These data will be published in a separate paper. In addition, we point out that insulin therapy can restore euglycemia and reduce glycogen accumulation in diabetic βV59M mice, which also supports the idea that the effects we see are due to elevated glucose and not K_{ATP} channel activation *per se*.

5. Another study that would be convincing of their hypothesis in the βV59M mouse model would be to isolate islets from these IGT and mild HG models and demonstrate that their function and transcriptional profiles could be restored after removal from hyperglycemia.

We respectfully point out that culture of isolated islets always runs the risk that the centre of the islets will become hypoxic due to lack of a blood supply, and thereby affect beta-cell metabolism. We therefore prefer to look at the effects of restoring euglycemia in vivo. We now show that changes in gene expression are almost fully reversed after 2 weeks of sulphonylurea therapy in mice that had been severely diabetic for 2 weeks (new Figure 10). We believe this answers the reviewer's concern.

6*. While the data in this manuscript are convincing, there are obvious limitations to both systems in the translation to human diabetes. Some confirmatory studies using human models or data would be highly supportive. Indeed, this could be studies in human islets/Endo bh-3/5 cells or alternatively comparison of their gene expression results with data from human islet donors with pre-diabetes/IGT (see for example Wigger, et al. Nature Metabolism PMID 34183850 or Bandesh, et al. bioRxiv 2025 PMID 39896672).*

We are not convinced that EndoC cells are the best model in this instance because they do not respond to high-glucose culture (in terms of altered insulin secretion) like primary human or mouse beta-cells (Even the recently developed GLTX-EndoCβh5 only become dysfunctional in response to chronic exposure to high palmitate plus high glucose, but not high glucose alone). We also feel that there are problems with isolated human islets when studying metabolism because of: (i) the long period between isolation and study; (ii) the fact the patient may have been treated with a cocktail of drugs before the islets were harvested; (iii) in the past we have found (using confocal microscopy) that the centre of the islets becomes necrotic and metabolism is impaired.

However, we have had reasonable results with human islet microspheres from Insphero. These are smaller in size and thus less prone to hypoxia. They can also be cultured at differe nt glucose concentrations. As shown in Reviewer Figure 1, culture at high gluco se concentrations leads to a glucose-dependent decline in insulin secretion, as it does in INS -1 cells. (Figure 1 has been redacted, see reply to Reviewer 1) Insulin content also decreas es markedly. See also the paper Mir-Coll et al (reference 12 in our paper).

We thank the reviewer for pointing us towards the paper by Wigger et al. They show that both *ALDOB* and proteins involved in mitochondrial metabolism show similar changes in human islets to those we see in mouse islets. They also report a very strong correction of *ALDOB* expression with HbA1c. This has now been now added to the paper.

Minor Comments:

1. Check for consistency in "INS-1 cells" (sometimes referred to in manuscript as INS1-cells)
Thank you. We have now ensured that INS-1 cells are correctly referred to throughout.

2. Figure 1A – any significant changes in the low glucose conditions? Looks like trending down for each subsequent group
We originally compared insulin secretion at 2 and 20mM glucose for each set of cells. We now present p values for comparisons between insulin secretion from cells cultured at 5mM glucose and at higher glucose concentrations when stimulated with 20mM glucose.

3. Figure 2A-D, line 160 – these genes are dramatically altered in 25G cells, but in 8G "dramatically" seems a bit of an overstatement for everything except perhaps Aldob. Also, Gapdh really doesn't look dramatically altered in any group. Please consider tempering the wording here.

This has now been rephrased. We now say "Many glycolytic/gluconeogenic genes were sensitive to mild chronic hyperglycaemia, including *Aldob, Gapdh, Pfkfb3 and Pfkfb2,* with *AldoB* and *Pfkfb2* being markedly altered even in 8G-cells (Fig.2a-d)."

4. Line 261 – the "A" in Acaca is not italicized
This has now been corrected

5. Line 262, 264, 269, 275 – add figure references at the end of the sentences; these are the only ones the reviewer noticed, but please check the rest of the text to ensure proper figure citing throughout.
This has now been corrected.

6. Very minor comment – when listing genes for both Figures 5 and 6, listing the genes in the text in the order they appear in the figures would make it easier for the reader to cross-check between the two.
This has now been done for Fig. 6. We feel it reads better to leave Fig.5 as it stands because some of the genes mentioned are only given in the Supplementary data files.

7. Figure 7B-K – Data points are varying sizes in each of the graphs and would look a little cleaner if they were consistent sizes
This has now been corrected.

8. Figure 7J also looks slightly vertically stretched compared to the other graphs in the figure
This has now been corrected.

9. Figure 8B&F and 8D&H – Data points and axis titles are different sizes and would look cleaner if they were consistent with each other
This has now been corrected.

10. Line 440 – change "beta cell" to "β-cell" for consistency
This has now been corrected

Reviewer Figure 1 a-b

Figure Redacted

Reviewer Figure 1 c-d

Figure Redacted

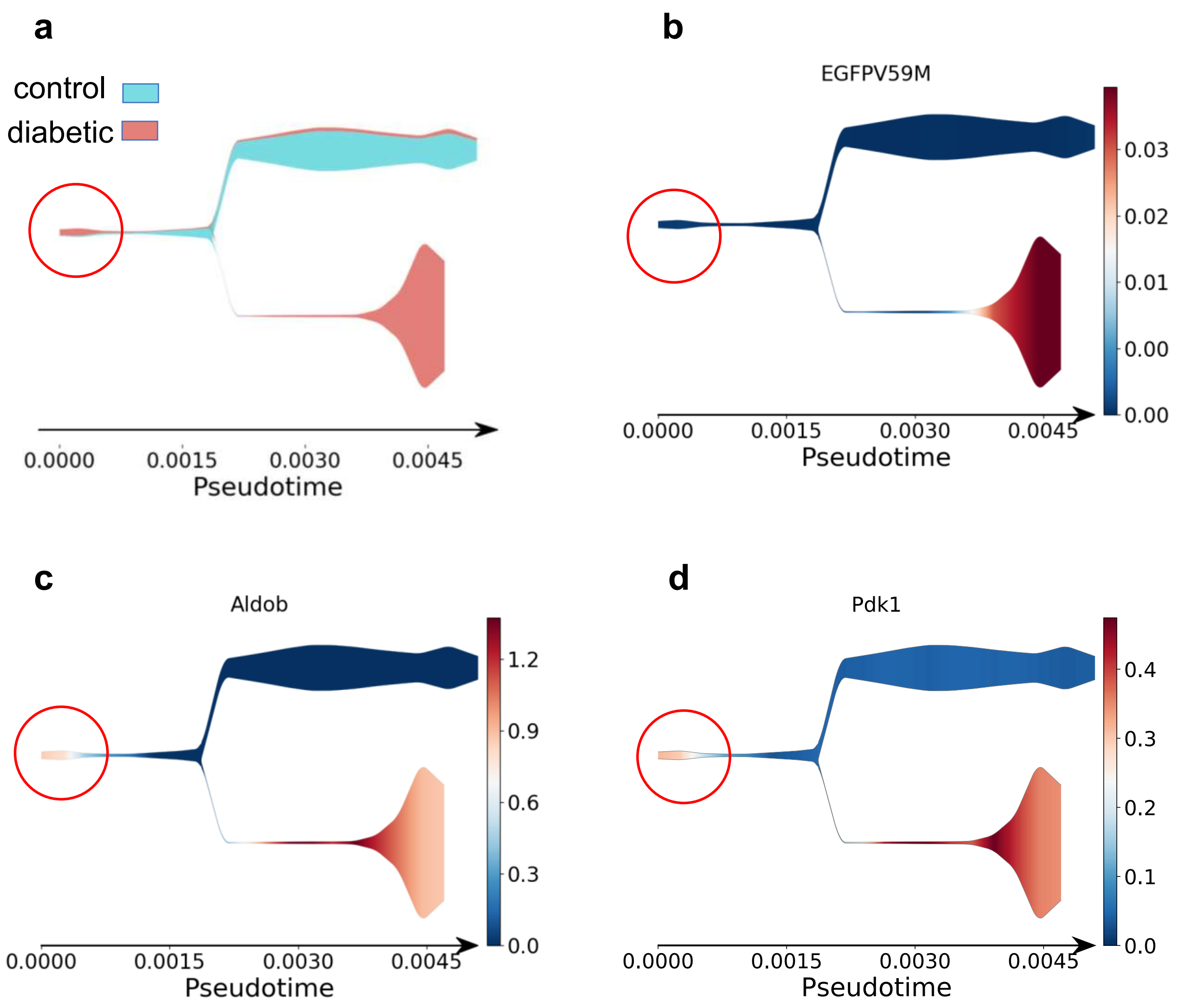

Reviewer Fig. 2. Pseudotime analysis of single beta-cell RNAseq from control and diabetic mice.

The colour code for relative expression is shown on the right with blue indicating low expression and red high expression. Red circles indicate beta-cells in diabetic mice that did not express the Kir6.2-V59M mutation

Panel A. Analysis of the full transcriptome differentiates control and diabetic beta-cells. Note that the top right-hand stream is primarily control cells and the lower right-hand stream is entirely diabetic cells. The left-hand stream shows a separate population of diabetic beta-cells. **Panel B** shows that this smaller population of beta-cells, like the control cells, did not express EGFP. EGFP lies downstream of the Kir6.2-V59M mutation in our construct and is co-expressed with the Kir6.2-V59M mutation. EGFP expression thus differentiates those beta-cells which express the Kir6.2-V59M mutation from those that do not. However, these beta-cells will still be exposed to the chronic hyperglycaemia – and as shown in **Panels C and D**, they show the same changes in glycolytic genes as cells in which EGFP is expressed: for example, AldoB and Pdk1 are increased in both diabetic beta-cells that express the V59M mutation (bottom right-hand stream) and those that do not (left-hand stream, circled in red).

We conclude that chronic hyperglycaemia not K_{ATP} channel activation causes changes in metabolic genes

Reply to the reviewers

We are delighted that our manuscript is provisionally publishable in *Nature Communications*. We have made the corrections suggested by the reviewers and the editors and our replies are as follows. We understand that these have already been accepted.

Reviewer 1.
Thank you for taking my advice and adding the data of the in vivo intervention study. Even though not all readouts were normalized (as expected in complex biological systems) I think this adds a nice "translational" angle to the in vitro-heavy story. One detail: Please make sure the number of animals is correctly outlined. New Figure 10 says 4,4,4 (legend) while the figure looks more like 4,3,4 (difficult to say). Suppl. Fig. 5 (derived from the same data I guess) states 4,3,4. If the number of samples are not the same, the authors should state why they excluded a dataset. I have no further questions/suggestions.

The reviewer is correct that there is a typo in the legend to Figure 10a. It should read 'severe-HG (red, n=3)'. i.e.

(a) PCA plot of RNAseq data from islets isolated from control (green, n=4), severe-HG (red, n=3) and glibenclamide-treated severe-HG (blue, n=4) mice. The plot is based on the 500 most variable genes across all samples.

We thank the reviewer for spotting this typo.

Figure 10b and Supplementary Figure 5 are correctly labelled.

Reviewer 2.
The authors adroitly addressed remaining concerns in the manuscript. I have no remaining concerns other than having the authors include the data for reviewer figure #1 which included studies in islet microspheres from Insphero as well as INS-1 cells with mannoheptulose into the manuscript. These data would likely fit nicely within Figure 10 of their manuscript and would add weight related to the inclusion of studies in human samples. I commend the authors on an excellent study.

We thank the reviewer for their kind comments on the paper. This reviewer suggested we include the data for the Reviewer Figure 1 that we supplied in response to his/her earlier comment. However, we respectfully point out that (as previously stated) this data is included in another paper (that is now under revision elsewhere) on the effects of both chronically enhanced and reduced glucokinase activity on insulin secretion. This makes it problematic to add here. This data was obtained and submitted following the submission of our manuscript to *Nature Communications*. We also think that it would be difficult to add to Figure 10 without making the panels extremely small, and that omission of this data does not materially detract from the manuscript which is primarily focussed on the effects of mild chronic hyperglycaemia and its reversal by restoration of euglycaemia.
